# Size-Transformable Nanoparticles for Tumor Drug Delivery: Distinct Roles of Shrinkage, Enlargement, and Reassembly

**DOI:** 10.3390/biomedicines14091971

**Published:** 2026-09-01

**Authors:** Kibeom Kim

**Affiliations:** Department of Chemistry and Life Science, Sahmyook University, Seoul 01795, Republic of Korea; kibumsy@syu.ac.kr

**Keywords:** size-transformable nanoparticles, tumor drug delivery, deep tumor penetration, nanoparticle assembly, nanoparticle disassembly, tumor microenvironment, multistage transformation

## Abstract

Although fixed-size nanoparticles have achieved substantial therapeutic success, a single fixed size may not be optimal for the competing requirements of stability in circulation, tumor accumulation, deep tumor penetration, cellular uptake, and intra-tumoral retention. This review frames this fixed-property limitation as the need to switch structural states in response to successive biological barriers and analyzes original research articles published from 2021 to 2025 on large-to-small transformation, small-to-large transformation and assembly, or multistage assembly-disassembly-reassembly. Rather than treating the stimulus or material as an analytical endpoint, the framework maps each pre-, intermediate-, and post-transition state to its biological site and barrier-specific functions. Direction, intermediate structure, site, sequence, and the temporal characteristics were evaluated together with coupled changes in charge, morphology, stiffness, and surface interactions. Large-to-small transformations generally shift the carrier function from tumor accumulation to penetration or uptake, whereas small-to-large transformations support retention, depot formation, or organelle-localized activity. Multistage systems assign distinct functions to the sequential structural states. However, direct in vivo structural evidence and physiologically relevant kinetic measurements have been limited. Future designs should, therefore, verify that the required structure forms at the intended site and time, and directly connect structural transitions with delivery and therapeutic functions.

## 1. Introduction

Cancer is a disease that is difficult to treat effectively, not only because of the biological complexity of tumor cells themselves, but also because of the heterogeneous vascular structure, dense extracellular matrix, high interstitial pressure, and immunosuppressive tumor microenvironment (TME) [1,2,3]. Anticancer chemotherapy is widely used in clinical practice; however, its therapeutic effect is limited owing to non-specific biodistribution, low tumor accumulation, toxicity to normal tissues, and drug resistance [4,5,6]. Various NP-based drug delivery systems have been developed [7,8,9]. Nanoparticles (NPs) have the advantage of improving the solubility and stability of poorly soluble drugs, enabling the simultaneous loading of multiple therapeutic and imaging agents, and targeting tumors or specific cells through surface functionalization [10,11,12]. However, unlike the excellent results reported in preclinical studies, the number of NPs delivered to the tumor is still limited, and uneven distribution within the tumor remains a major reason for preventing clinical use [13,14,15].

For systemically administered NPs to reach tumor cells, they must overcome a series of biological barriers, including maintaining stability in the blood, avoiding clearance by the immune and mononuclear phagocyte systems, extravasating across the tumor vasculature, diffusing through the tumor interstitium, crossing the cell membrane, and releasing the drug intracellularly [16,17,18]. However, the physicochemical properties of NPs required for each delivery step differ or are conflicting [19,20,21]. Particle size is a major factor that determines blood circulation, tumor accumulation, tissue penetration, cellular uptake, and systemic clearance [22,23,24]. In general, relatively large NPs have a high drug loading capacity and structural stability and are advantageous in reducing rapid clearance from the blood; however, it is difficult for them to pass through the dense tumor stroma and reach tumor cells far from the blood vessels [25,26,27]. In contrast, small NPs exhibit high diffusivity and tissue penetration but may be cleared through the kidneys or liver before they can sufficiently accumulate in the tumor or leak rapidly even after reaching the tumor [28,29,30]. Because size acts together with shape, stiffness, surface charge, ligand presentation, material composition, and protein adsorption, there are fundamental limitations in simultaneously optimizing blood circulation, tumor accumulation, deep tumor penetration, and long-term retention with a single fixed-size NP. [31,32,33].

### 1.1. Rationale, Mechanisms, and Advantages of Structural Transformation

To address this size-dependent delivery tradeoff, size-transformable NPs have attracted increasing attention [34,35]. These systems are designed to maintain an initial state suited to circulation and tumor accumulation and then alter their dimensions or morphology in response to endogenous stimuli, including acidic tumor pH, hypoxia, reducing conditions, reactive oxygen species, and overexpressed enzymes, or to exogenous stimuli such as light and heat [36,37,38,39,40,41,42,43,44]. Unlike conventional stimulus-responsive systems that primarily trigger cargo release, structural transformation can also modify nanocarrier diffusion, surface interactions, cellular uptake, tumor retention, and subcellular distribution [45,46].

Three general transformation trajectories address different stages of tumor delivery. Large-to-small systems use an initially stable structure for circulation and tumor accumulation and subsequently release smaller units to improve stromal penetration or cellular access [47,48,49,50,51]. Small-to-large systems begin with diffusible particles or precursors that form aggregates, depots, nanofibers, or networks at the target site, thereby promoting local or organelle-level retention [52,53,54,55,56,57,58,59,60,61,62,63]. Multistage systems combine sequential disassembly and reassembly so that distinct structural states perform barrier-specific functions in different biological compartments [64,65,66]. Thus, the value of shape- or size-shifting behavior lies not in structural change alone but in matching the direction, site, sequence, and kinetics of each transition to a specific delivery barrier [67,68,69].

### 1.2. Scope and Analytical Framework

Existing reviews have organized size-transformable nanotherapeutics based on transformation direction, stimulus, material composition, or transformation mechanism [34,35,48,50,52]. The present framework adds a distinct analytical step; it reconstructs the structural-state trajectory and assigns pre-transition, intermediate, and post-transition states to the biological compartment and barrier-specific functions in which each state operates. Thus, the two systems activated by the same stimulus do not need to occupy the same functional category and similar size changes may be interpreted differently when they occur extracellularly, intracellularly, or within an organelle. The reported transformation times were retained only under the experimental conditions and were not treated as directly comparable kinetic constants unless the primary study reported such parameters.

This review examines the recent developments in size-transformable NPs for tumor drug delivery, focusing on original research articles published between January 2021 and December 2025. Fourteen therapeutic platforms met the core scope. One imaging-only hydrophobic core-tunable ultra-pH-sensitive nanoprobe (HUNP) study was retained separately as a diagnostic mechanistic comparator because it directly demonstrated lysosomal disassembly and functional signal activation [70]. We analyzed large-to-small transformations, small-to-large aggregation or morphological transformations, and multistage trajectories in which sequential states address different biological barriers.

The central objective was to establish barrier-adapted structural switching as a design principle, rather than claiming that size alone determines delivery. The systems were evaluated according to their direction, intermediate structure, biological site, sequence, reported temporal observations, evidence strength, and final function, together with coupled changes in charge, morphology, stiffness, protein corona, and composition. The drug loading and administered doses are reported in the corresponding platform descriptions when available. As transformable and control formulations are not always fully matched in terms of loading, surface chemistry, ligand density, or stimulus responsiveness, the independent contribution of size transformation cannot be precisely quantified in every study. Nevertheless, the reported improvements in penetration, retention, uptake, release, and therapeutic response support the meaningful contribution of structural transformation to the overall treatment outcome.

To improve transparency, we conducted a targeted narrative search for original studies published between January 2021 and December 2025 using PubMed, Web of Science, Scopus, and Google Scholar. Search concepts combined “size-transformable nanoparticle,” “size-shrinkable nanoparticle,” “size-enlarging nanoparticle,” “nanoparticle aggregation,” “nanoparticle disassembly,” “in situ assembly,” “morphology transformation,” “tumor,” “cancer,” “penetration,” and “retention.” Studies were included if they reported an experimentally characterized change in particle dimensions, aggregation state, or extended morphology in a tumor-related model and connected the transition to delivery, imaging, or therapeutic function. Reviews, conference abstracts, duplicate reports, non-tumor applications, and systems showing drug release or charge reversal without an experimentally supported structural transition were excluded. The reference lists of relevant reviews were also screened. Because this is a targeted narrative rather than a systematic review, the selection emphasizes mechanistic relevance and primary structural evidence. Fourteen therapeutic studies formed the principal evidence set; the HUNP imaging study was retained only as a separately labeled diagnostic mechanistic comparator.

In this review, size transformation refers to a measured change in the hydrodynamic dimension or spatial extent that is expected to alter transport, retention, uptake, or release. Aggregation refers to the association between pre-existing particles; self-assembly to the organization of molecular or nanoscale building blocks; and morphological transformation to a change in shape, aspect ratio, or topology, such as a sphere-to-fiber transition; these processes may coexist [71,72]. “Large” and “small” are therefore treated as literature-informed and context-dependent rather than as universal binary categories. Dimensions near 100 nm and below 20 nm provide useful reference points for accumulation and stromal penetration, respectively. However, the shape, charge, stiffness, protein corona, tumor architecture, and measurement method shift these relationships [22,23,24,34,35]. Platforms outside these reference ranges were classified based on their within-platform structural trajectories and functions, which were experimentally investigated in the original study.

**Scheme 1:**
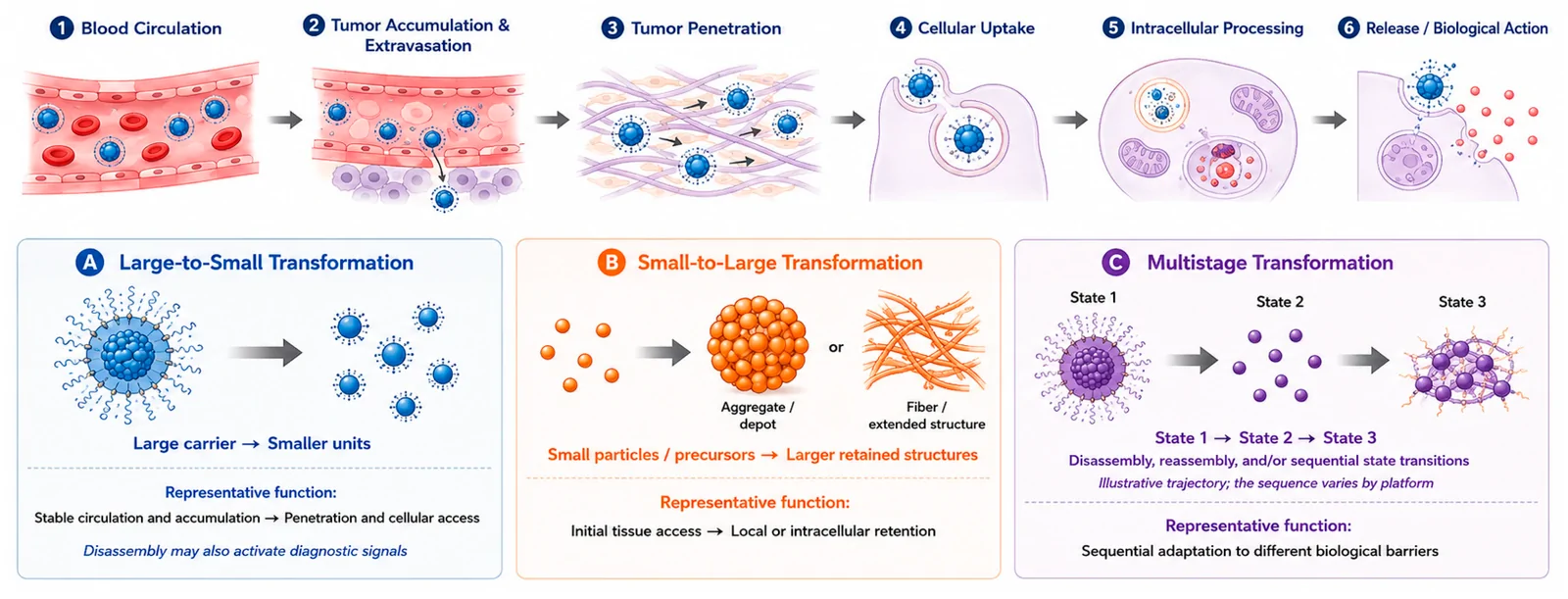
Barrier-adapted structural switching of size-transformable nanoplatforms during tumor delivery. Nanoplatforms encounter sequential barriers from blood circulation and tumor accumulation to tissue penetration, cellular uptake, intracellular processing, and biological action. Three representative strategies are shown: (**A**) large-to-small transformation for enhanced penetration and cellular access; (**B**) small-to-large transformation for local or intracellular retention; and (**C**) multistage transformation for sequential adaptation to different biological barriers.

A system was considered unidirectional when one dominant structural transition determined its principal delivery function, even when shell shedding, charge reversal, ligand exposure, and drug release occurred simultaneously. A system is considered multistage when two or more sequentially resolved structural states perform different barrier-specific functions at different sites or times. This functional rule distinguishes unidirectional systems with coupled physicochemical changes from multistage systems with sequentially functionally differentiated structural states. Scheme 1 illustrates the six successive stages of tumor delivery, from blood circulation and tumor accumulation/extravasation to stromal penetration, cellular uptake, intracellular processing, drug release, and biological action, and highlights the functional advantages of nanoplatform size transformation at the corresponding stages.

## 2. Large-to-Small Transformation

Large-to-small transformation is a strategy to resolve the size-dependent trade-off, in which relatively large nanostructures are advantageous for blood circulation and tumor accumulation, whereas small particles are advantageous for diffusion and cellular uptake into the dense tumor stroma [72,73]. This system is designed to maintain a stable initial structure during systemic circulation and release smaller NPs, micelles, dendrimers, polymer unimers, or molecular components in response to tumor-associated or external stimuli after reaching the tumor [34,35,48]. Therefore, size reduction is not a simple structural collapse but a process that switches the primary carrier function from circulatory stability and tumor accumulation to deep tumor penetration, cellular uptake, and intracellular activation [34,35,48].

Large-to-small transformations are induced by various mechanisms such as shell degradation, cleavage of stimulus-cleavable linkers, weakening of supramolecular interactions, micelle disintegration, and cluster dissociation [37,48]. Size reduction occurring in the tumor extracellular space primarily enhances diffusion through the extracellular matrix (ECM) and access to cancer cells distant from blood vessels, whereas degradation occurring in endosomes or lysosomes contributes more directly to cytoplasmic drug release or imaging signal activation [35,48]. Additionally, in some systems, surface charge reversal or targeted ligand exposure occurs along with size reduction, simultaneously increasing cell-membrane interactions and endocytosis [74]. Therefore, even for the same large-to-small transformation, the biological function may vary according to the transformation site and accompanying changes in surface properties [34,35,74].

In this section, we compare recent nanoplatforms that ultimately decrease in size in response to stimuli, such as exogenous light and heat, acidic pH of the tumor, reactive oxygen species, hypoxia, and matrix metalloproteinases. This includes not only systems in which the initial particles are directly shrunk, but also multistage shrinkage in which ultrasmall particles are emitted through temporary enlargement and carrier disassembly. In particular, the analysis focuses on the correlation between the size of the initial and post-transformation structures, the biological compartment in which the conversion occurs, the time required for the change, direct evidence of deep tumor penetration, and therapeutic efficacy. Through this comparison, we discuss how large-to-small transformation may address the size-dependent delivery trade-off between tumor accumulation and penetration and how size reduction is linked to drug release, surface charge change, and phototherapy to realize a sequential delivery function.

### 2.1. Extracellular Disassembly and Shell Shedding for Penetration

Wu et al. developed NIR-responsive upper critical solution temperature (UCST)-type nanomicelles to sequentially utilize the characteristics of large NPs, which are advantageous for tumor accumulation, and small NPs, which are advantageous for deep tumor penetration [43]. This system consisted of thermoresponsive p(AAm-co-AN)-PEG-LA, the anticancer drug doxorubicin (DOX), and magnetic gold nanoflower (Fe_3_O_4_@AuNF). The final Fe_3_O_4_@AuNFs/DOX-M showed an initial size of approximately 150 nm.

p(AAm-co-AN)-PEG-LA micelles showed a UCST of approximately 43 °C, and when the temperature reached UCST, the particle size decreased to approximately 5 nm. When 808 nm NIR irradiation was applied to the tumor area 24 h after intravenous injection, the Au nanoflowers converted light into heat and raised the tumor temperature above the UCST. As a result, the micelles were converted into ultrasmall structures, promoting DOX release. In vivo, the tumor temperature reached approximately 51 °C after 5 min of NIR irradiation; however, the kinetics of the structural transitions were not directly measured. DOX loading (12.5 ± 5.7%) and encapsulation efficiency (70.3 ± 5.9%) were reported, and the DOX-containing treatment groups received the same nominal DOX dose (5 mg/kg). Although photothermal heating and triggered drug release also contribute to treatment response, the enhanced penetration and efficacy observed after irradiation support the meaningful contribution of size transformation to the overall therapeutic outcome.

This large-to-small transformation was designed to convert the delivery function of the initial nanomicelles that accumulated in the tumor into deep tissue penetration and cellular uptake. In cell experiments, cellular uptake of Fe_3_O_4_@AuNFs/DOX-M increased from 30.93 ± 5.0% after 1 h to 70.54 ± 9.3% after 12 h, and the DOX fluorescence signal in MCF-7 cells after NIR irradiation was approximately 2.2 times higher than that of the non-irradiated group. This indicates that NIR-triggered size transformation can improve the intracellular delivery of drugs.

In the tumor penetration experiment, 24 h after the second nanomicelle administration, the tumor was irradiated with NIR light for 5 min, and the distribution of DOX in the tumor tissue was analyzed 4 h later. In the group without NIR irradiation, DOX was mainly distributed around the tumor blood vessels, whereas in the irradiated group, it spread to avascular regions away from the blood vessels. DOX fluorescence in this area was approximately six times higher than that in the non-irradiated group, indicating that size change and drug release improved drug distribution in deep tumors.

Fe_3_O_4_@AuNF acted as a photothermal agent that induced NIR-responsive size transformation, while providing photothermal therapy and computed tomography/magnetic resonance imaging (CT/MRI) functions. As a result of using Fe_3_O_4_@AuNFs/DOX-M in combination with NIR irradiation in the MCF-7 xenograft model, a tumor inhibition rate of 92.6% and complete tumor disappearance in three out of five mice were observed. On this platform, NIR irradiation sequentially induced size reduction, DOX release, and photothermal effects. Therefore, this study provides an example of a strong antitumor effect achieved by linking NIR-triggered size transformation to deep tumor drug distribution, cellular uptake, and chemo-photothermal therapy. The efficacy experiment used five mice per group.

Zhang et al. developed CPIM, an NIR-triggerable ROS-responsive cluster-bomb nanoplatform, to sequentially utilize the tumor accumulation ability of large NPs and the deep tumor penetration ability of small NPs [44]. CPIM was prepared by crosslinking PI, a PAMAM dendrimer loaded with IR780, and PM, a PAMAM dendrimer loaded with 1-methyl-tryptophan (1-MT), an indoleamine 2,3-dioxygenase (IDO) inhibitor, with a thioketal (TK) linker, and coating them with chondroitin sulfate (ChS). The sizes of PI and PM were 8.58 and 9.15 nm, respectively, and the final CPIM showed a size of approximately 135.43 nm and a surface charge of −25.63 mV.

Systemically administered CPIM was designed to accumulate in the tumor and then change stepwise due to hyaluronidase (HAase) and high ROS concentrations in the TME. First, HAase decomposes the ChS coating to convert the surface charge of CPIM from negative to positive, and then ROS cuts the TK linker, decomposing the cluster of approximately 135 nm into PI and PM bomblets of approximately 10 nm in size. Additionally, upon 808 nm NIR irradiation, IR780 generated additional ROS, promoting the large-to-small transformation of CPIM and drug release.

The released small PAMAM bomblets moved to the depths of the tumor through passive diffusion and caveolae-mediated transcytosis, owing to size reduction. In the B16F10 multicellular tumor spheroid, IR780 was distributed to the center of the spheroid after NIR irradiation, and in an experiment using an excised tumor, the penetration depth of the irradiated group was 4036.9 μm, which was more than 10 times greater than the 325.2 μm of the non-irradiated group. In B16F10 tumor tissues in vivo, IR780 was mainly distributed around blood vessels in the non-irradiated group; however, in the NIR-irradiated group, it spread to tumor areas away from the blood vessels.

IR780 simultaneously induces photodynamic therapy (PDT) and photothermal therapy (PTT) by NIR irradiation. When 808 nm NIR was irradiated to the tumor area for 5 min 12 h after administration of the NPs, the tumor temperature of the CPI group, a single IR780-loaded control formulation similar to CPIM, increased by 17.7 °C compared to before irradiation, and was estimated to have reached approximately 50–55 °C based on thermal imaging, showing a photothermal effect superior to the temperature increase of 8.0 °C in the free IR780-treated group. In addition, PDT and PTT with IR780 induced immunogenic cell death, and 1-MT inhibited the IDO pathway, converting the immunosuppressive TME into an immune-activating environment.

As a result of using CPIM and NIR in combination in the B16F10 melanoma model, the tumor volume was maintained at 1.23 times the size before treatment on the 14th day of treatment, while it increased to 6.80 times in the free IR780 and NIR combination group, 15.17 times in the CPIM group without NIR, and 19.61 times in the normal saline group, indicating that the CPIM and NIR combination group had the strongest primary tumor growth inhibition effect. This treatment activated systemic antitumor immunity by increasing dendritic cell maturation and the ratio of CD4^+^ helper T cells to CD8^+^ cytotoxic T cells, and decreasing regulatory T cells. An abscopal effect was observed in tumors that were not directly irradiated with NIR light, and lung metastasis was effectively suppressed. In summary, ROS-responsive large-to-small transformations serve as an integrated treatment strategy that improves deep tumor penetration and links PDT, PTT, and IDO pathway inhibition, and antitumor immune responses. Primary tumor growth and tumor weight analyses were performed using five mice per group, whereas immune cell analyses used n = 3.

Meng et al. developed polyethylene glycol-poly L-lysine-dimethylmaleic anhydride/mannose–polyethylenimine–cytosine-phosphate-guanine oligodeoxynucleotide (PPD/MPC), a pH-responsive adjuvant micelle nanosystem for deep tumor penetration and simultaneous improvement of the immunosuppressive TME [75]. PPD/MPC comprises a negatively charged polyethylene glycol-poly L-lysine-dimethylmaleic anhydride (PEG–PLL–DMMA; PPD) shell electrostatically complexed with a positively charged mannose-conjugated polyethylenimine (MA–PEI) core carrying a cytosine–phosphate–guanine oligodeoxynucleotide (CpG ODN; MPC). At physiological pH 7.4, the PPD shell shields the positively charged MPC core, maintaining a particle size of approximately 98 nm and a surface charge of −7.82 mV, thereby increasing stability in the blood and reducing non-specific cellular absorption. On the other hand, at pH 6.8, which simulates a slightly acidic TME, the PPD shell was separated due to decomposition of the DMMA bond; the particle size decreased from 97.9 ± 9.2 nm to 52.3 ± 5.4 nm, and the surface charge changed from −7.82 mV to +23.29 mV. Therefore, the size transformation of this system can be defined as charge reversal accompanied by shrinkage due to acid-triggered shell shedding.

The MPC core, whose size was reduced, penetrated deep into the tumor owing to improved tissue diffusion. In B16F10 melanoma multicellular spheroids, PPD/MPC was mainly distributed in the periphery at pH 7.4. However, at pH 6.8, it reached the center of the spheroid after 4 h of treatment and was widely distributed throughout the entire spheroid after 12 h. In vivo, the spatial separation of the PPD shell and MPC core was observed 2 h after intravenous administration. While the separated PPD shell mainly remained in the area adjacent to the tumor blood vessels and the tumor periphery, the MPC core of approximately 52 nm penetrated deep into the tumor away from the blood vessels. Subsequently, the positively charged MPC core was selectively absorbed by tumor-associated macrophages (TAMs), tumor-infiltrating dendritic cells (TIDCs), and myeloid-derived suppressor cells (MDSCs) through mannose–cluster of differentiation (CD) 206 (CD206) receptor interactions and polyethylenimine (PEI)-mediated electrostatic adsorption. Thus, size reduction is responsible for deep tumor penetration at the tissue level, while charge reversal and mannose targeting improve delivery to immunosuppressive cells at the cellular level. The in vivo formulations were administered at the same CpG dose (5 mg/kg) to reduce dose-related confounding factors. The observed spatial separation, deep penetration, and immune effects supported the meaningful contribution of size reduction, whereas differences in shell composition, mannose presentation, charge behavior, and disassembly capability also contributed to the overall response.

In PPD/MPC, CpG ODN and PEI were introduced as adjuvant ingredients instead of cytotoxic anticancer drugs. CpG ODN activates intracellular Toll-like receptor (TLR) 9 (TLR9) signaling, and PEI stimulates the TLR4 pathway. The combined immunomodulatory action of the two components converts M2-type TAMs into an antitumor M1 phenotype, reprograms MDSCs, and promotes the maturation. Accordingly, the expression of the immunosuppressive factors interleukin (IL)-10 (IL-10) and transforming growth factor (TGF) beta (TGF-β) decreased, while the expression of immune-activating factors such as L-12, tumor necrosis factor alpha (TNF-α), and interferon gamma (IFN-γ) increased. In vivo, M1-type TAMs, mature dendritic cells, and CD8^+^ cytotoxic T lymphocytes increased, whereas regulatory T cells and MDSCs decreased, thereby converting the TME to an immune-activated state by simultaneously re-educating the three types of immunosuppressive cells.

In the B16F10 melanoma model, PPD/MPC showed the strongest tumor growth and lung metastasis inhibition effects among the comparative formulations, and the survival rate at 40 days after treatment was 66.7%. In the tumor rechallenge experiment, unlike the tumor recurrence rate of 83.3–100% in other treatment groups, the recurrence rate in the PPD/MPC group was reduced to 33.3%, and long-term antitumor immune memory was confirmed through an increase in effector memory T cells. This study shows that shell shedding-mediated shrinkage induced in a slightly acidic TME improves deep tumor penetration, and that simultaneous charge reversal and mannose targeting promote delivery to immunosuppressive cells, which can lead to the inhibition of tumor growth, metastasis, and recurrence. Therefore, PPD/MPC is an example in which shrinkage goes beyond simply improving the delivery of drugs to tumor cells and is used to simultaneously target and reprogram multiple immunosuppressive cells present deep within the tumor. In this system, shell shedding reduces the particle size while exposing the positively charged surface and mannose targeting moiety. These linked physicochemical changes sequentially improve deep tumor infiltration and cellular uptake by immunosuppressive cells, strengthening the antitumor immune response. Six biologically independent animals per group were used for the in vivo efficacy study.

Wang et al. developed a CuS-DOX@MA/Gel nanocomposite that sequentially responded to matrix metalloproteinase (MMP)-2 and acidic pH [76]. In this platform, DOX was bound to copper sulfide NPs (CuS NP) modified with polyethyleneimine through an acid-cleavable imine bond, and the outside was coated with a gelatin shell containing 3-methyladenine (3-MA), an autophagy inhibitor. The initial nanocomposite (approximately 180.1 nm) was designed to accumulate in tumors while maintaining structural stability and drug retention during circulation and to convert into smaller structures after reaching the tumor. The primary study described DOX loading determination and normalized major treatment groups by CuS dose; however, complete parity in DOX loading, 3-MA content, surface charge, and gelatin-shell properties across all controls was not established. Nevertheless, the observed enhancement in stromal penetration supports the meaningful contribution of MMP-2-responsive shrinkage, while the overall efficacy reflects the combined action of composition, irradiation, and triggered release.

MMP-2, which is overexpressed in the tumor ECM and around blood vessels, degrades the outer gelatin shell and reduces the size of CuS-DOX@MA/Gel from 180.1 nm to about 57.2 nm. This large-to-small transition represents enzyme-triggered removal of the degradable outer shell rather than uniform shrinkage of the entire particle, thereby exposing the smaller CuS-DOX-based particles inside. Transformation occurs in the tumor extracellular space and switches the dominant delivery function from tumor accumulation by the larger initial structure to tissue penetration by the smaller exposed structure.

Particles of approximately 57.2 nm produced by MMP-2 passed through the tumor stroma more effectively than the initial nanocomposite. In the 4T1 multicellular tumor spheroid, particles in the absence of MMP-2 mainly stayed at the periphery of the spheroid, but after MMP-2 treatment, strong fluorescence was observed at depths of 50, 75, and 150 μm. Even in tumor tissues, size-reduced particles were distributed beyond the periphery of blood vessels and into deep regions, indicating that size reduction in this system is not a simple structural change but a key process that switches the delivery function from tumor accumulation to deep tumor penetration.

Decomposition of the gelatin shell led to size reduction and the simultaneous local release of 3-MA. In the presence of MMP-2, approximately 72% of 3-MA was released within 20 min and approximately 80% was released within 6 h, whereas in the absence of MMP-2, only approximately 2% was released over 6 h. The released 3-MA inhibits class III phosphatidylinositol 3-kinase, blocks protective autophagy in cancer cells, and increases sensitivity to DOX. Subsequently, when small CuS-DOX particles enter the cell, the imine bond is cleaved in the acidic environment of the lysosome, releasing the DOX. Therefore, this platform is a sequential reaction system that spatially separates shrinkage–penetration–3-MA release by MMP-2 in the tumor extracellular space and DOX release by the intracellular acidic environment.

CuS NPs functioned not only as drug carriers but also as phototherapeutic agents that responded to 808 nm NIR irradiation. The heat and ROS generated by laser-irradiation-induced PTT and PDT, respectively, were combined with DOX chemotherapy and 3-MA-mediated autophagy inhibition. In the 4T1 tumor-bearing mouse model, the group using CuS-DOX@MA/Gel in combination with NIR irradiation showed the strongest tumor inhibition effect, with a tumor inhibition rate of 83.9%, and no significant weight loss or histological damage to the major organs was observed. The antitumor experiments were performed using six mice per group. In summary, this system enhances the efficacy of multiple treatments by linking the enhancement of tumor penetration due to MMP-2-responsive shrinkage with 3-MA-mediated autophagy inhibition, DOX chemotherapy, and NIR-triggered phototherapy.

### 2.2. Diagnostic Mechanistic Extension: Intracellular Disassembly for Signal Activation

**Figure 1:**
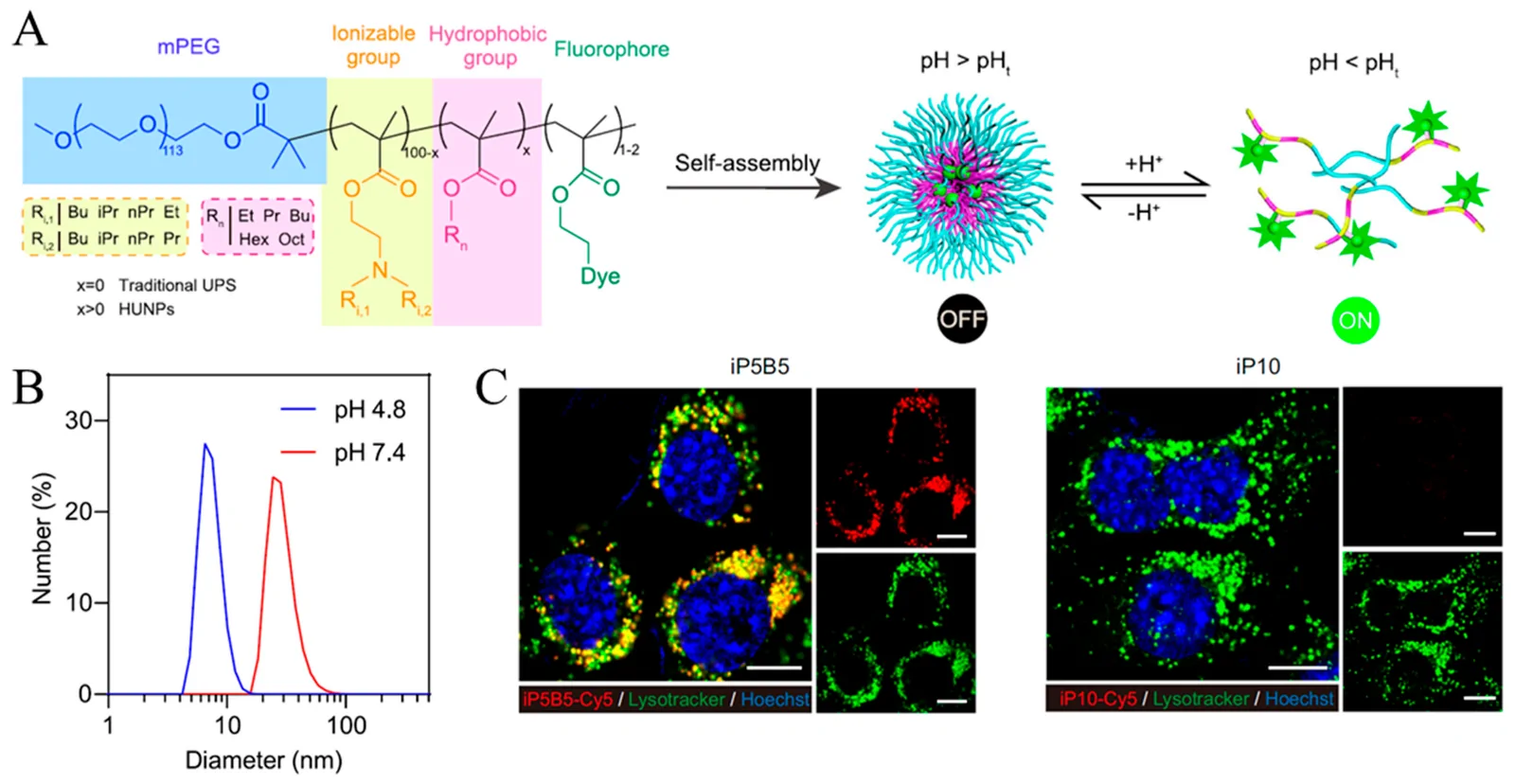
Core-composition-dependent disassembly and intracellular fluorescence activation of HUNPs. (**A**) Design and pH-responsive activation mechanism of hydrophobic core-tunable ultra-pH-sensitive NPs (HUNPs). HUNPs remain fluorescence-quenched in the micellar state at pH values above their transition pH (pH_t_) but disassemble into fluorescent unimers following protonation at pH values below pH_t_. (**B**) Hydrodynamic size distributions of iP5B5 at pH 7.4 and 4.8, showing its transformation from approximately 30 nm NPs to approximately 8 nm unimers. (**C**) Intracellular activation and lysosomal localization of iP5B5-Cy5 and iP10-Cy5 in 4T1 tumor cells. Red, Cy5-labeled nanoprobes; green, LysoTracker; blue, Hoechst. Adapted with permission from Ref. [70]. Copyright © 2024 The author(s), published by Springer Nature.

Pan et al. developed a HUNP to improve the sensitivity of tumor imaging and the accuracy of image-guided surgery by linking the cellular uptake of NPs and intracellular pH-responsive disassembly (Figure 1A) [70]. Existing ultra-pH-sensitive nanoprobes can selectively activate fluorescence in acidic organelles; however, only approximately 15–20% of the NPs accumulated in tumors are internalized by cancer cells. Therefore, even if sufficient tumor accumulation is achieved, the diagnostic signal may not be sufficiently activated because of the limited number of particles that reach the acidic intracellular environment. Instead of increasing total tumor accumulation, the authors proposed a strategy to increase cell membrane binding and intracellular uptake of particles that have already reached the tumor by controlling the composition of the hydrophobic core and then amplifying the fluorescence signal through lysosomal disassembly.

HUNP are core–shell micelles with a size of approximately 30 nm, consisting of a hydrophilic methoxy poly(ethylene glycol) (mPEG) shell and a hydrophobic core containing an ionizable tertiary amine. The authors produced nanoprobe libraries with different core hydrophobicities by adjusting the ratio of the ionizable monomer, 2-(diisopropylamino)ethyl methacrylate (iDPA-MA), to the non-ionizable monomer, butyl methacrylate (BMA) or 2-hydroxyethyl methacrylate (HEMA). The optimized iP5B5 had a composition of mPEG114-b-P(iDPA50-r-BMA50) and maintained a low fluorescence in the OFF state at physiological pH through homotypic Förster resonance energy transfer (homo-FRET) and self-quenching between fluorophores. When iP5B5 was introduced into the cell and exposed to the acidic environment of endosomes and lysosomes, the micelles became unstable due to cooperative protonation of tertiary amines, and the approximately 30 nm structure was decomposed into approximately 8 nm polymer unimers at pH 4.8 (Figure 1B). In this process, homo-FRET and self-quenching were resolved, and the fluorescence signal under acidic conditions increased by more than 100 times that of the micellar OFF state.

Although the outer mPEG shell was the same, increasing the hydrophobicity of the core markedly enhanced cell membrane binding and cellular uptake. Compared to the conventional iP10 nanoprobe, iP5B5 containing 50% BMA increased its binding to 4T1 cells by approximately 10.3-fold, intracellular uptake by approximately 8.7-fold, and confocal fluorescence intensity by approximately 22.3-fold. Consistent with these results, iP5B5-Cy5 exhibited substantially stronger intracellular fluorescence activation and lysosomal co-localization than iP10-Cy5 (Figure 1C). Of the total NPs present in the tumor, the percentage that entered the cell increased from approximately 15–20% to a maximum of approximately 50%, but there was no significant difference in the total tumor accumulation measured with the Always-ON probe. Therefore, the improved imaging signal can be interpreted as the result of improved cell membrane binding and cellular uptake due to increased core hydrophobicity, rather than an increase in total tumor accumulation and the subsequent pH-responsive disassembly and fluorescence activation that occurred in the lysosome. iP5B5 showed strong tumor-selective fluorescence in 4T1, CT26, MC38, and MCF-7 tumor models and in the KrasLSL-G12D/+; Trp53LSL-R172H/+; Pdx1-Cre (KPC) pancreatic cancer model. The contrast-to-noise ratio was approximately 157.7, which was higher than approximately 26.9 for iP7HE3, enabling the identification of tumor boundaries and small lesions and image-guided tumor resection.

HUNP is not a therapeutic drug delivery platform and is therefore presented separately as a diagnostic mechanistic comparator. This demonstrates that intracellular micelle disassembly can switch function toward lysosomal fluorescence activation rather than stromal penetration or therapeutic release. 

The key characteristics of the large-to-small transformation platforms discussed in this section, including their structural trajectories, activation sites, delivery functions, tumor models, and reported in vivo evidence, are summarized in Table 1.

**Table 1:** Large-to-small transformation platforms for tumor therapy and diagnostic imaging.

Platform/Type	Size/Structural Trajectory	Trigger/Site	Delivery Function/Cargo/Modality	Model	Reported Time/In Vivo Evidence	Ref.
Fe_3_O_4_@AuNF micelle (hybrid NP)	~150 nm micelle → ~5 nm micelles; UCST; micelle disassembly	NIR heat/extracellular tumor	Deep tumor penetration and cellular uptake; DOX; chemo/PTT; CT/MRI; Efficacy n = 5	MCF-7	Not directly measured; NIR 5 min, in vivo heating; Evidence: Indirect	[43]
CPIM (polymer cluster)	135 nm cluster → <10 nm NPs; TK cleavage; disassembly	ROS/ extracellular TME	Deep penetration and immune activation; IR780 + 1-MT; PDT/PTT; Tumor efficacy n = 5; immune assays n = 3	B16F10	Not directly quantified; ROS/NIR-triggered; Evidence: Indirect	[44]
PPD/MPC (polymer micelle)	97.9 nm → 52.3 nm NPs; Shell shedding; charge reversal	pH 6.8/ extracellular TME	Tumor penetration and immune-cell uptake; CpG ODN; immunotherapy; Efficacy n = 6	B16F10	Separation observed 2 h after dosing, in vivo; Evidence: Indirect	[75]
CuS-DOX@MA/Gel (hybrid NP)	180.1 nm → 57.2 nm NPs; Gelatin-shell degradation	MMP-2/ extracellular TME	Tumor penetration and cellular uptake; DOX + 3-MA; PDT/PTT; Efficacy n = 6	4T1	MMP-2-associated release: 20 min–6 h, in vitro; structural rate NR; Evidence: Indirect	[76]
HUNP (polymer micelle)	30 nm micelle → 8 nm unimer; Protonation; disassembly	Acidic pH/endosome–lysosome	Cellular uptake and lysosomal signal activation; Fluorophore; imaging; Diagnostic study; n = 3–5 animals depending on endpoint;	4T1; CT26; MC38; MCF-7; KPC	pH-dependent state comparison; formal rate NR; Evidence: Indirect (diagnostic)	[70]

Note: Reported times are study-specific observations under the stated conditions and are not directly comparable kinetic constants. Direct evidence denotes structural visualization in tumor tissues, whereas fluorescence retention or functional changes alone are classified as indirect evidence. NR, not reported. Endpoint-specific sample sizes are shown only when identifiable in primary reports. The observed therapeutic effects should not be attributed exclusively to size transformation when drug loading, surface charge, ligand density, composition, or stimulus conditions do not fully match between comparison groups. Abbreviations: 1-MT, 1-methyltryptophan; 3-MA, 3-methyladenine; AuNF, gold nanoflower; CPIM, formulation designation used in the original study; CpG ODN, cytosine-phosphate-guanine oligodeoxynucleotide; CT, computed tomography; CuS, copper sulfide; DOX, doxorubicin; HUNP, hydrophobic core-tunable ultra-pH-sensitive nanoprobe; KPC, KrasLSL-G12D/+; Trp53LSL-R172H/+; Pdx1-Cre pancreatic cancer model; MMP, matrix metalloproteinase; MPC and PPD, formulation designations used in the original study; MRI, magnetic resonance imaging; NIR, near-infrared; NP, nanoparticle; NR, not reported; PDT, photodynamic therapy; PTT, photothermal therapy; ROS, reactive oxygen species; TK, thioketal; TME, tumor microenvironment; UCST, upper critical solution temperature.

## 3. Small-to-Large Size and Morphology Transformations

Small-to-large transformation is a strategy in which small NPs or molecular precursors that are advantageous for tumor penetration are converted into larger aggregates, nanofibers, microscale depots, or polymeric networks after reaching the target site [52,71]. The small initial structure facilitates extravasation and diffusion through the tumor extracellular matrix, but its high mobility may limit local retention and promote back diffusion or cellular exocytosis [52]. Increasing the size or aspect ratio after reaching the tumor may limit interstitial backflow, lymphatic drainage, and cellular exocytosis, prolonging the local residence time and drug exposure [52]. Therefore, the key to this approach is not to administer a large structure systemically but to approach the tumor while maintaining high initial mobility and then convert to a less mobile structure only at the desired location [52].

Small-to-large transformations can be induced by tumor-related enzymes such as furin or matrix metalloproteinase, acidic pH, bioorthogonal reactions, and intracellular apoptotic enzymes [53,71]. Aggregation and nanofiber formation in the tumor extracellular space mainly contribute to tumor retention and local drug depot formation [52,53]. By contrast, intracellular aggregation or polymerization can inhibit exocytosis, concentrate therapeutic agents within specific organelles, induce physical or functional organelle damage, and regulate cell death [59,71,77]. In particular, the transition from spherical NPs to high-aspect-ratio nanofibers needs to be evaluated as a separate shape transition because, unlike a simple increase in size, it can change the molecular arrangement, intracellular location, and biodegradability [77].

In this section, we analyze recent small-to-large systems involving the aggregation of individual NPs, enzyme-guided nanofiber reassembly, bioorthogonal drug depot formation, and intracellular polymerization. Comparisons of each system focus on whether the initial structure contributes to invasion, whether enlargement directly increases tumor or intracellular retention, and how the final structure is linked to local retention, organelle damage, and subsequent therapeutic functions. We also discuss the major design challenges that small-to-large transformations must address, such as the capture of the tumor periphery due to excessively rapid assembly, in vitro release due to slow conversion, and the possibility of long-term accumulation of large nondegradable structures. For clarity, Section 3.1 groups systems whose principal transition is particle-to-aggregate enlargement or covalently crosslinked drug-depot formation, whereas Section 3.2 separately discusses sphere-to-fiber morphology transformation, lysosomal nanofiber assembly, and intracellular polymerization. The biological site of each transition was retained as an independent descriptor, rather than as a subsection boundary.

### 3.1. Particle Aggregation and Drug-Depot Formation

Xie et al. developed AuNPs-D&H-R&C, a furin-responsive gold NP (AuNP) system, to simultaneously overcome the low tumor retention of NPs and drug release from cancer cells in DOX-resistant breast cancer [78]. Here, D&H represents DOX and hydroxychloroquine (HCQ), and R&C represents RK peptide and 2-cyano-6-aminobenzothiazole (CABT), respectively. This platform consisted of AuNPs-D and H-RK bound to a furin-cleavable RK peptide (RVRRCK), AuNPs-D&H-CABT bound to CABT, and DOX and HCQ were co-loaded at a mass ratio of 1:6. The initial particles of approximately 38 nm were designed to maintain a dispersed state suitable for circulation in the blood and tumor access and to be converted into large aggregates through covalent bonds between particles after reaching the tumor.

Size conversion is initiated by furin, a pro-protein convertase that is overexpressed in breast cancer. When furin cleaves the RVRR region of the RK peptide, the 1,2-aminothiol group of the terminal cysteine is exposed, causing cycloaddition with the cyano group of CABT introduced into adjacent particles. As this reaction was repeated between multiple particles, individual AuNPs were connected to a covalent aggregate. Therefore, the RK peptide acts as a substrate that recognizes tumor enzymes and exposes reactive aminothiols, while CABT functions as a complementary reactive group that forms crosslinks between particles.

The hydrodynamic diameter of AuNPs-R&C before furin treatment was 38.2 ± 1.2 nm, but gradually increased over time, reaching 263.2 ± 10.6 nm after 48 h. On the other hand, PEG-modified control AuNPs only increased from 36.7 ± 0.9 nm to 52.1 ± 2.4 nm. 24 h after furin treatment, a structure in which several AuNPs were connected to each other was observed by transmission electron microscopy (TEM); in the ultraviolet–visible absorption spectrum, the gold surface plasmon absorption shifted from approximately 520 nm to 640 nm. These results show that size transformation does not occur immediately but is a continuous aggregation process that takes place over several to tens of hours.

Furin-triggered aggregation performs different functions depending on its location. Aggregates formed in the tumor extracellular milieu limit the reverse diffusion of NPs from the tumor stroma into the bloodstream and increase local retention. Aggregates consisting of dozens of AuNPs were observed within the cells, and the increased particle size inhibited exocytosis and improved the intracellular accumulation of NPs and drugs. AuNPs-D&H-R&C reduced DOX efflux by approximately 8.7 times compared to free DOX and inductively coupled plasma–optical emission spectrometry analysis also showed that the intracellular gold content of RK/CABT-functionalized AuNPs was higher than that of the PEG-modified control. In other words, extracellular enlargement was mainly responsible for interstitial retention and intracellular enlargement was mainly responsible for cellular retention.

In the accumulated particles, the linkers that reacted with the acidic environment were cleaved, releasing DOX and HCQ. The 24-h DOX release rate was 13.8% at pH 7.4 but increased to 72.1% and 86.8% at pH 6.5 and 5.5, respectively. While DOX induced DNA damage, HCQ inhibited lysosomal function and protected the autophagic flux, re-sensitizing DOX-resistant MCF-7/ADR breast cancer cells to DOX. Additionally, HCQ repolarized M2-like macrophages to an M1-like phenotype by activating nuclear factor kappa B (NF-κB) signaling. The apoptotic cell rate of the AuNPs-D&H-R&C treatment group was 49.3%, which was higher than 14.3% of the non-aggregating AuNPs-D&H-P and 23.4% of the AuNPs-D-R&C loaded with DOX alone.

In MCF-7/ADR breast tumor-bearing nude mice, AuNPs-D&H-R&C showed higher tumor fluorescence and photoacoustic (PA) signals and a stronger tumor growth inhibition effect than the PEG-modified control. In tumor tissues, along with increased apoptosis, increased expression of CD86, an M1-associated marker, and decreased expression of CD206, an M2-associated marker, were confirmed, supporting macrophage repolarization. Overall, this study shows that dispersed AuNPs are converted into aggregates by furin in the extracellular and cellular milieu of tumors and that this location-dependent enlargement increases tumor retention and intracellular accumulation, which can help overcome drug resistance and modulate the immune microenvironment. Key pharmacokinetic and imaging comparisons were performed using five mice per group. The primary study assessed tumor accumulation and therapeutic response within the experimental treatment window, but did not directly quantify long-term degradation, organ-level clearance, excretion, or persistence of furin-induced gold aggregates; therefore, short-term imaging and efficacy do not establish clearance of the transformed AuNP structure.

Cao et al. developed a bioorthogonal in situ assembly system that formed a microscale drug depot in the extracellular space of the tumor to solve the problem of nanomedicines reaching the tumor being removed quickly and not maintaining a sufficient drug concentration [57]. This platform consists of two types of spherical NPs, D-NPs and C-NPs, approximately 65 nm in size. D-NPs were prepared from cysteine–polyethylene glycol-block-polylactic acid (DA-Cys-PEG-b-PLA) masked with 2,3-dimethylmaleic anhydride (DA), and C-NPs were made from 2-cyanobenzothiazole–polyethylene glycol-block-polylactic acid (CBT-PEG-b-PLA). The two types of particles were designed to retain independent NPs in the blood, move to the tumor, and then covalently bind to each other after extravasation to transform into large extracellular aggregates.

Size conversion is initiated by the slightly acidic extracellular pH of the tumor. At physiological pH 7.4, the cysteine on the surface of D-NP is shielded by DA and does not react with C-NP; however, at pH 6.5, the acid-labile amide bond is cut, and the free 1,2-aminothiol group of cysteine is exposed. This functional group undergoes bioorthogonal cycloaddition with the cyano group of CBT present on the surface of the C-NPs, forming a firefly luciferin-like structure. As this reaction is repeated between adjacent D-NPs and C-NPs, the particles are crosslinked via covalent bonds. Therefore, D-NP functions as a tumor acidity-responsive cysteine donor and C-NP functions as a complementary CBT reaction partner that binds to cysteine.

Dynamic light scattering (DLS) and TEM analyses showed that both D-NPs and C-NPs had similar initial sizes and spherical morphologies of approximately 65 nm. The mixture of two particles showed little change in size over 24 h at pH 7.4, but at pH 6.5, the number and size of aggregates increased over time, reaching a hydrodynamic diameter of 2611 ± 115 nm after 24 h. Confocal laser scanning microscopy also observed microscale aggregates after 2 h of incubation. Additionally, cysteine–CBT cycloaddition and the formation of covalent bonds between particles were confirmed through fluorescence spectroscopy, gel permeation chromatography (GPC), and proton nuclear magnetic resonance (^1^H NMR) analysis. On intravital imaging, large aggregates were observed in the tumor tissue 6 h after intravenous administration, and the formed structure was maintained even after 48 h, supporting in vivo transformation and long-term retention.

Size transition in this system occurs in the tumor extracellular milieu and is aimed at enhancing local retention and sustained drug release rather than cellular uptake. Initially, 65 nm NPs can pass through tumor blood vessels and move into the extracellular space, but after being converted into aggregates of approximately 2.6 μm, they are difficult to back-diffuse into the blood vessels or lymphatic system and function as an extracellular drug depot. The blood pharmacokinetics and major organ distribution of D-NP/C-NP were similar to those of the non-assembled control. However, differences in accumulation within the tumor were evident 24 h after administration. After 96 h of administration, the tumor fluorescence of D-NP/C-NP was approximately 2.6 times higher than that of the control, and the drug content in the tumor increased for 48–96 h when quantified using a platinum prodrug. This shows that the difference in retention after assembly, rather than the initial tumor delivery, determines the final tumor accumulation. The supporting information reported closely matched DOX loading for the paired carriers (4.89 ± 0.45% in D-NP and 4.76 ± 0.51% in S-NP), and the in vivo regimens were dose-normalized by cargo. This reduces loading-related confounding, and enhanced tumor retention and accumulation support the contribution of structural assembly to therapeutic outcomes, whereas differences in terminal chemistry and stimulus responsiveness remain relevant.

The authors evaluated the therapeutic utility of a drug depot using batimastat (BB94), which inhibits extracellular MMPs as a representative extracellular-targeting drug. In an orthotopic 4T1 murine breast tumor model, BB94-loaded D-NPs/C-NPs inhibited the expression and activity of MMP-2, MMP-3, and MMP-9 by maintaining the drug in the tumor extracellular space for a long period. Compared with the phosphate-buffered saline (PBS) control, the number of pulmonary metastatic nodules was reduced by 93%, and primary tumor growth was also suppressed. Additionally, this platform was able to keep multiple drugs together at the same tumor site by loading DOX, the indoleamine 2,3-dioxygenase inhibitor NLG919, and the colony-stimulating factor 1 receptor inhibitor BLZ945 onto different particles. Chemoimmunotherapy with this cocktail resulted in a tumor inhibition rate of 82.49% and reduced lung metastasis. Quantification of major tumor and immune cells in the D-NP/C-NP study was performed using n = 5. The primary study visualized intratumoral aggregates from 6 to 48 h, quantified tumor fluorescence up to 96 h, and monitored efficacy or survival for 27–48 days, but did not directly determine the degradation products, excretion routes, or clearance of the covalently crosslinked microdepots.

After combination treatment, cytotoxic CD8^+^ T cells and M1-like macrophages in the tumor increased, while regulatory T cells and CD11b^+^CD206^+^ M2-like macrophages decreased. In addition, the inhibition of the IDO pathway by NLG919 and macrophage reprogramming by BLZ945, combined with DOX-induced antitumor immunity, alleviated the immunosuppressive TME. Overall, this study shows that dispersed NPs of approximately 65 nm are converted into covalent aggregates of approximately 2.6 μm over 2–24 h in response to the slightly acidic pH of the tumor extracellular milieu, and that this change can lead to extracellular retention, sustained drug release, and simultaneous delivery of multiple drugs.

**Figure 2:**
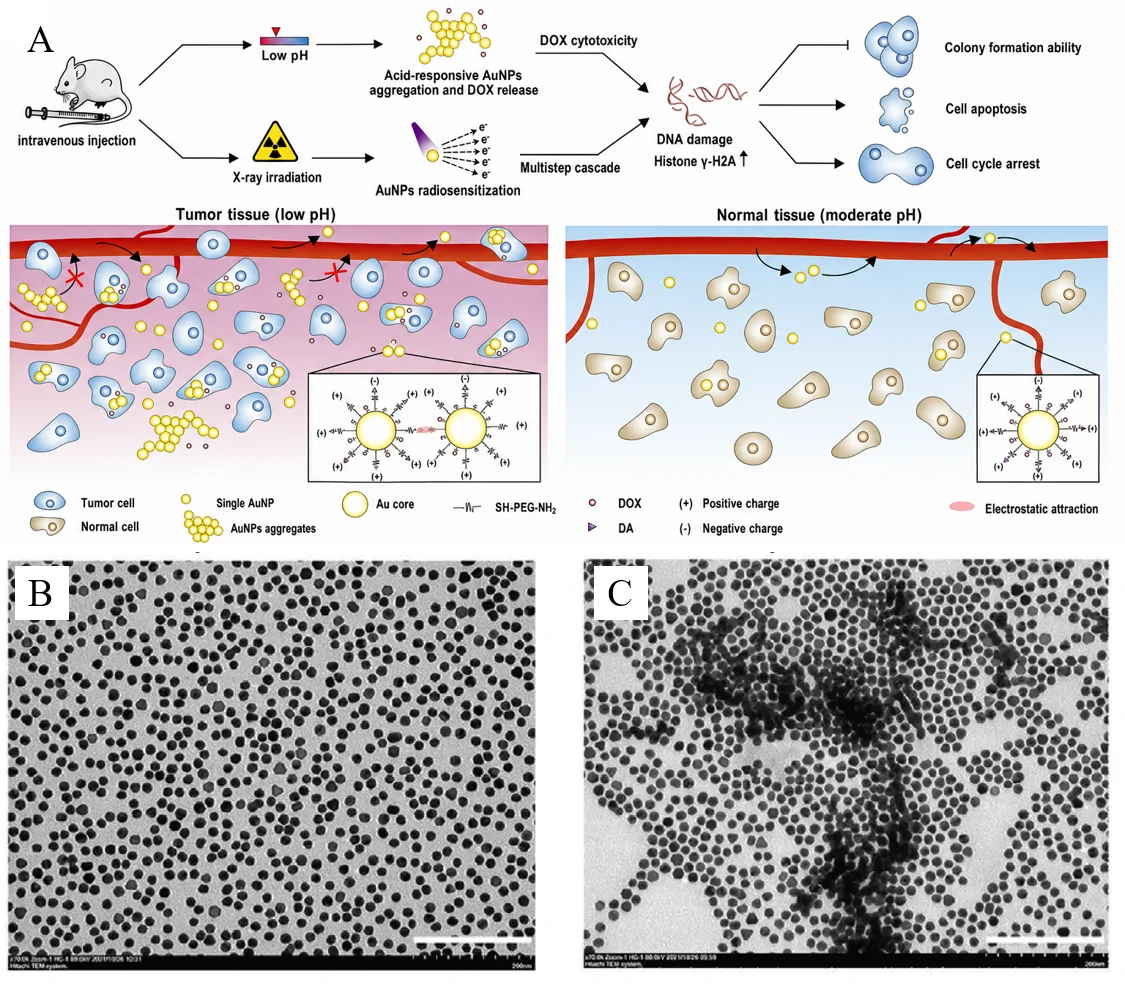
Tumor acidity-induced aggregation of AuNPs-P-DA for combined chemotherapy and radiosensitization. (**A**) Schematic illustration of the selective aggregation of AuNPs-P-DA and DOX release in the acidic tumor microenvironment, resulting in enhanced AuNP-mediated radiosensitization and DOX-induced cytotoxicity. (**B**) Transmission electron microscopy (TEM) image of dispersed AuNPs-P-DA after incubation at pH 7.4 for 1 h. (**C**) TEM image showing AuNP aggregation after incubation at pH 6.5 for 1 h. Scale bars = 200 nm. Adapted with permission from Ref. [55]. Copyright © 2022 Wiley-VCH GmbH.

Luan et al. developed AuNPs-D-P-DA, an acid-responsive aggregatable nanosystem, to solve the problem of limited radiosensitization in esophageal cancer due to low intratumoral AuNP accumulation [55]. This platform introduces DOX, PEG, and 2,3-dimethylmaleic anhydride (DA) into an Au core of approximately 21.39 nm, and the hydrodynamic diameter after surface modification was approximately 48.82 nm. The Au core acts as an intrinsic radiosensitizer that increases X-ray energy deposition and ROS production, whereas DOX functions as a chemotherapeutic cargo bound through an acid-labile hydrazone linker. The initially small particles were designed to maintain stability in circulation and tumor accessibility, and were then converted into large aggregates after reaching the tumor to simultaneously enhance radiosensitization and chemotherapy (Figure 2A).

Aggregation begins when the dimethylmaleic amide bond at the end of PEG is hydrolyzed in the slightly acidic environment of the tumor. At physiological pH, the amine at the end of PEG is shielded by the negatively charged DA, thereby maintaining electrostatic repulsion and PEG-mediated steric stabilization between the particles. In contrast, at pH 6.5, DA was partially removed, and the protonated primary amine was exposed, forming both positive and residual negative regions on the particle surface. Accordingly, electrostatic attraction occurs between oppositely charged regions of adjacent particles, and several AuNPs assemble into noncovalent aggregates. Therefore, this system corresponds to a small-to-large transformation that is aggregated by acid-triggered partial charge reversal rather than covalent crosslinking between the particles.

The hydrodynamic diameter of AuNPs-P-DA increased from 50.4 nm to 420.6 nm after 4 h at pH 6.5, whereas it changed only from 52.3 nm to 75.9 nm at pH 7.4. TEM images obtained after 1 h showed that AuNPs-P-DA remained dispersed at pH 7.4 (Figure 2B), whereas distinct aggregates were observed at pH 6.5 (Figure 2C), confirming the acid-induced small-to-large transformation. The resulting aggregates were not readily redispersed after the restoration of the neutral pH, indicating that the structural transformation was largely irreversible. Because the average particle size remained at approximately 48 nm in serum-containing conditions at pH 7.4, premature aggregation in the bloodstream was considered limited. However, because the in vitro transformation experiment was conducted in a constant pH 6.5 buffer, the transformation kinetics and final aggregate size may differ in the spatially heterogeneous pH environment of actual tumors.

Aggregates perform different functions depending on their location in the extracellular and cellular milieu of a tumor. Small AuNPs that escaped from tumor blood vessels formed aggregates in the acidic extracellular microenvironment. In tumor tissue images, these structures were mainly distributed around blood vessels. Large aggregates inhibit backflow into the bloodstream due to high tumor interstitial pressure, increased intratumoral gold concentration, and retention time. Particles introduced into cells also aggregated in acidic endosomes/lysosomes, increased particle size, limited exocytosis, and improved intracellular AuNP and DOX accumulation. In in vivo TEM, aggregatable AuNPs were observed as aggregates inside tumor tissues and cells, whereas non-aggregating control AuNPs were dispersed as individual particles.

The acidic environment simultaneously controlled DOX release and AuNP aggregation. The 24-h cumulative DOX release rates were 13.0% at pH 7.4, 27.2% at pH 6.5, and 63.0% at pH 5.5, indicating that DOX was partially released from the tumor extracellular space, mainly from the more acidic endosome/lysosome. AuNP aggregates accumulated in the tumor enhanced radiation-induced DNA damage by increasing X-ray absorption and local energy deposition. DOX acted synergistically with the radiation effect by interfering with DNA replication and generating free radicals. In KYSE-510 esophageal cancer cells, the combination of AuNPs-D-P-DA and 3 Gy radiation increased phosphorylated histone H2AX (γ-H2AX) signal, comet tail, G2/M cell-cycle arrest, and apoptosis and most strongly inhibited colony formation. The apoptosis rates of aggregateable AuNPs-P-DA and AuNPs-D-P-DA were 32.87% and 66.57%, respectively, which were higher than the 9.29% apoptosis rate of the radiation-only group and the corresponding non-aggregating formulations. For the cited in vitro efficacy data, colony formation quantification was performed using n = 3, whereas the n for the flow cytometric apoptosis percentages was not explicitly identified in the corresponding legend.

In KYSE-510 human esophageal cancer xenograft-bearing mice, AuNPs-D-P-DA showed higher fluorescence and photoacoustic (PA) tumor signals than non-aggregating AuNPs-D-P, and the strongest tumor growth inhibition, tissue necrosis, and apoptosis were observed in the treatment group combining DOX and radiation. Body weight, liver/kidney function, and histological examination of major organs showed no significant systemic toxicity. In summary, this study showed that AuNPs of approximately 50 nm were converted into aggregates of approximately 421 nm within 4 h in response to the acidic pH of the tumor extracellular and cellular milieu and that this enlargement can enhance AuNP-mediated radiosensitization and DOX-based chemotherapy by inhibiting interstitial backflow and cellular exocytosis. The primary study reported biodistribution and evaluated blood biochemistry and major organ histology at the end of the 28-day treatment, but did not directly quantify the degradation, excretion, or long-term organ persistence of the acid-induced gold aggregates.

### 3.2. Shape/Morphology Transformation and In Situ Polymerization

Cao et al. developed DOX/AGLR, a peptide nanocarrier that converts spherical particles into nanofibers in response to MMP-2, to compensate for the short residence time of small NPs, which is advantageous for tumor penetration [79]. AGLR is an amphiphilic peptide composed of a tumor-targeting arginine–glycine–aspartic acid (RGD) peptide, an MMP-2-sensitive GLIG sequence, and a hydrophobic domain. AGLR self-assembles in a physiological environment to form spherical NPs tens of nanometers in size. The NPs maintained their spherical morphology after DOX was loaded into the hydrophobic core. The initial NPs are responsible for the migration and penetration into the tumor tissue, and the high-aspect-ratio nanofibers formed after reaching the tumor are designed to increase the local retention of the drug. Although matched DOX loading between DOX/AGLR and non-transformable DOX/AGGG control has not been explicitly reported, the enhanced cellular uptake, tumor retention, and therapeutic response observed with DOX/AGLR support the meaningful contribution of MMP-2-induced nanofiber formation. However, its independent effect could not be precisely quantified because the differences in the peptide sequence, drug association, and surface charge were not fully controlled.

Shape changes begin when MMP-2, which is overexpressed in the tumor extracellular milieu, cleaves the GLIG sequence of AGLR. High-performance liquid chromatography (HPLC) and matrix-assisted laser desorption/ionization time-of-flight mass spectrometry (MALDI-TOF-MS) analysis revealed a new cleavage product and a hydrophobic LIGKRGD-NH_2_ fragment at *m*/*z* 757.4 after MMP-2 treatment. Enzyme cleavage changed the hydrophilic-hydrophobic balance and intermolecular interactions of the peptide, and the β-sheet structure increased in circular dichroism analysis. Accordingly, the cleaved peptide fragments were rearranged to convert low-aspect-ratio spherical NPs into high-aspect-ratio nanofibers. In contrast, AGGG, a control peptide without an MMP-2-sensitive sequence, maintained its spherical structure even after treatment with the same enzyme, supporting enzyme specificity of the conversion.

Morphological transformation was not completed immediately but progressed gradually over 12–72 h. TEM showed that elongation of spherical NPs began 12 h after MMP-2 treatment, nanofibrous structures increased at 24 h, and most particles were converted to nanofibers after 72 h. DLS also showed that the apparent hydrodynamic size increased to the micrometer range after enzyme treatment; however, because the final structure is a non-spherical nanofiber, this value is difficult to interpret as an accurate fiber length. Atomic force microscopy (AFM) showed that the initial NP height was approximately 10–15 nm, and after MMP-2 treatment, elongated fibers of lower height were observed. Thus, it can be confirmed that morphology-dependent reassembly occurred because of enzyme cleavage rather than simple particle aggregation of NPs.

Depending on the transition position, the initial and final nanofibers perform complementary delivery functions. In the tumor extracellular milieu, small spherical NPs diffuse into the tumor interior through the ECM, and the nanofibers formed by MMP-2 are not easily removed from the tumor owing to their low mobility and high aspect ratio. In the cellular experiments, DOX/AGLR showed higher cellular uptake and long-term intracellular DOX fluorescence than free DOX and non-transformable DOX/AGGG. In addition, DOX/AGLR lowered the expression of P-glycoprotein (P-gp), which is involved in drug excretion, and prolonged the intracellular retention of DOX bound to the nanofibers.

In H22 murine hepatocellular carcinoma-bearing mice, AGLR loaded with the NIR dye 1,1′-dioctadecyl-3,3,3′,3′-tetramethylindotricarbocyanine iodide (DiR) maintained a strong tumor signal even after 72 h of administration. The concentration of DiR in the tumor was 3.2 and 2.1 times higher than that of free DiR and non-transformable DiR/AGGG, respectively, and the tumor inhibition rate of DOX/AGLR was 77.1%, which was higher than 56.6% of free DOX and 59.1% of DOX/AGGG. In the lung metastasis model, the number of metastatic nodules in the DOX/AGLR group was 14.4% lower than that in the saline group. Simultaneously, the expression of MMP-2, C–C motif chemokine ligand 2, and CD31, which are related to epithelial–mesenchymal transition, angiogenesis, and metastasis, decreased, while the expression of E-cadherin, an epithelial marker, increased. The animal number for the cited tumor weight and metastatic nodule efficacy endpoints was not identifiable in the main article or available supporting information; therefore, these percentages are presented as reported point estimates. The primary study followed tumor-associated fluorescence for 72 h and evaluated efficacy and organ histology during the treatment period but did not directly measure degradation products, excretion, or persistence of the MMP-2-induced peptide nanofibers beyond that window.

DOX/AGLR not only increases drug accumulation but also regulates the immunosuppressive TME. After treatment, cluster of differentiation 80 and 86 double-positive (CD80^+^CD86^+^) dendritic cells increased, while CD4^+^ forkhead box P3-positive (CD4^+^Foxp3^+^) regulatory T cells decreased. Additionally, tumor necrosis factor-α (TNF-α) and IL-12 increased, while TGF-β and programmed death-ligand 1 (PD-L1) decreased. Taken together, this study shows that spherical NPs of tens of nanometers are reassembled into high-aspect-ratio nanofibers by MMP-2 over 12–72 h in the tumor extracellular milieu and that this shape conversion can link initial tumor penetration and subsequent long-term retention, thereby reducing tumor growth, lung metastasis, and immunosuppression.

To address the limited cancer cell selectivity of existing pyroptosis inducers, Zhang et al. developed NP-NH-D5, a tandem-responsive NP that is sequentially activated in the tumor extracellular matrix and intracellular lysosomes [63]. This NP is a spherical NP of approximately 60 nm in size in which F-C6-NH_2_, a non-peptide amphiphile, and F-SS-NH-GALGLP-D5, which contains an MMP-2-cleavable GALGLP-D5 sequence and a disulfide bond, are co-assembled at a molar ratio of 5:1. F-C6-NH_2_ consists of hydrophobic 3,5-bis(trifluoromethyl)benzene, a C6 alkyl chain, an amino-oxyluciferin fluorophore, and a polyethylene glycol linker containing a primary amine. Initially, NP-NH-D5 was designed to exhibit a weak negative charge of −4.39 ± 1.52 mV to reduce non-specific interactions with normal cells and maintain a stable particle state in the blood.

The first conversion occurs via MMP-2, which is overexpressed in the extracellular milieu of the tumor. When MMP-2 cleaves the GALGLP sequence of F-SS-NH-GALGLP-D5 and removes the negatively charged GLP-D5 fragment, NP-NH-D5 of approximately 60 nm is converted to NP-NH_2_ of approximately 55 nm. During this process, the particle size does not change significantly, but the zeta potential increases to +25.58 ± 1.40 mV, resulting in negative-to-positive charge reversal. NP-NH_2,_ converted to a positive charge, interacts strongly with the negatively charged cancer cell membrane, thereby increasing endocytosis. Therefore, rather than direct nanofiber formation, the extracellular MMP-2 response functions as an activation step that distinguishes normal cells from cancer cells and delivers the precursors necessary for intracellular transformation to lysosomes.

The second conversion is induced by glutathione (GSH) and γ-interferon-inducible lysosomal thiol reductase (GILT) present in tumor-cell lysosomes. When these reducing agents cleave the disulfide bond inside NP-NH_2_, the F-SS-NH-GAL fragment is removed and F-C6-NH_2_ remains. The released F-C6-NH_2_ is reassembled into β-sheet-rich nanofibers through hydrophobic interaction, π–π stacking, hydrogen bonding, and van der Waals interaction. In vitro disulfide reduction was completed within 2 h of GSH or GILT treatment. Fibrous structures began to appear at 30 min, and long extended nanofibers were formed after 6 h. The average diameter of the final nanofiber was approximately 12 nm and the height was approximately 3 nm, and the average Young’s modulus was 464 ± 75 MPa, showing high mechanical rigidity.

The formed nanofibers induced mechanical stress and a proton sponge effect on the lysosomal membrane owing to their high stiffness and abundant primary amines. As a result, lysosomal membrane permeabilization occurs and cathepsin B is released into the cytosol, activating the NOD-, LRR-, and pyrin domain-containing protein 3 (NLRP3) inflammasome and caspase-1. Activated caspase-1 induces pyroptosis by cleaving full-length gasdermin D (GSDMD-FL) into a pore-forming gasdermin D N-terminal fragment (GSDMD-N). In 4T1 cells, F-C6-NH_2_ was colocalized with lysosomes after 30 min of treatment, and long nanofibers and severe lysosomal damage were observed after 4 h. This cell death was accompanied by the release of IL-1β, IL-18, high-mobility group box 1, and adenosine triphosphate (ATP), leading to strong immunogenic cell death.

Mechanical stiffness is directly implicated in the biological activity of the NP-NH-D5 system. AFM measurements showed Young’s moduli of 634 ± 78 and 565 ± 93 MPa for the assembled nanofibers under the reported experimental conditions, respectively. The authors attributed lysosomal membrane permeabilization to the combined effects of nanofiber stiffness, mechanical stress, and the proton-sponge effect arising from the abundant primary amines [63]. Therefore, this study provides a direct example of how a transformation-induced change in morphology and mechanical properties contributes to biological activity. However, this mechanism cannot be generalized to other transformable platforms unless their mechanical properties are directly measured.

In orthotopic 4T1 breast tumor-bearing mice, NP-NH-D5 showed higher tumor accumulation than the MMP-2-inert control, and nanofiber formation and lysosomal disruption were directly observed in the bio-TEM images of the tumor tissue 24 h after administration. NP-NH-D5 inhibited primary tumor growth and lung metastasis; however, some tumors grew again after treatment. As a result of the combined use of PD-L1 antibody (anti-PD-L1), the orthotopic 4T1 tumor was completely removed in three out of five animals. Combination treatment increased dendritic cell maturation and CD8^+^ cytotoxic T-cell infiltration and decreased regulatory T cells and M2-like tumor-associated macrophages. In addition, by increasing the number of central memory T cells and effector memory T cells, a long-term immune memory is formed to suppress recurrence after tumor reinoculation.

In the late-stage 4T1 lung metastasis model, the combination of NP-NH-D5 and anti-PD-L1 decreased lung bioluminescence by approximately 31.9-fold at 36 days, and 3 of the 5 animals survived for more than 90 days. In the aggressive orthotopic Pan02 pancreatic tumor model, the combination treatment suppressed tumor growth and distant metastasis and extended the median survival from approximately 20 days in the PBS-treated group to approximately 82 days. In summary, this study demonstrates a position- and order-dependent nanofiber reassembly strategy in which a negatively charged NP of approximately 60 nm is activated into a positively charged particle of approximately 55 nm by MMP-2 in the tumor extracellular milieu and then reassembled into a long nanofiber with a diameter of approximately 12 nm by GSH and GILT in the tumor-cell lysosome. The primary study included major organ histology on day 21 and blood count and serum biochemistry measurements through day 28 in healthy mice; however, it did not directly quantify the degradation products, excretion routes, or long-term persistence of lysosomally assembled nanofibers.

**Figure 3:**
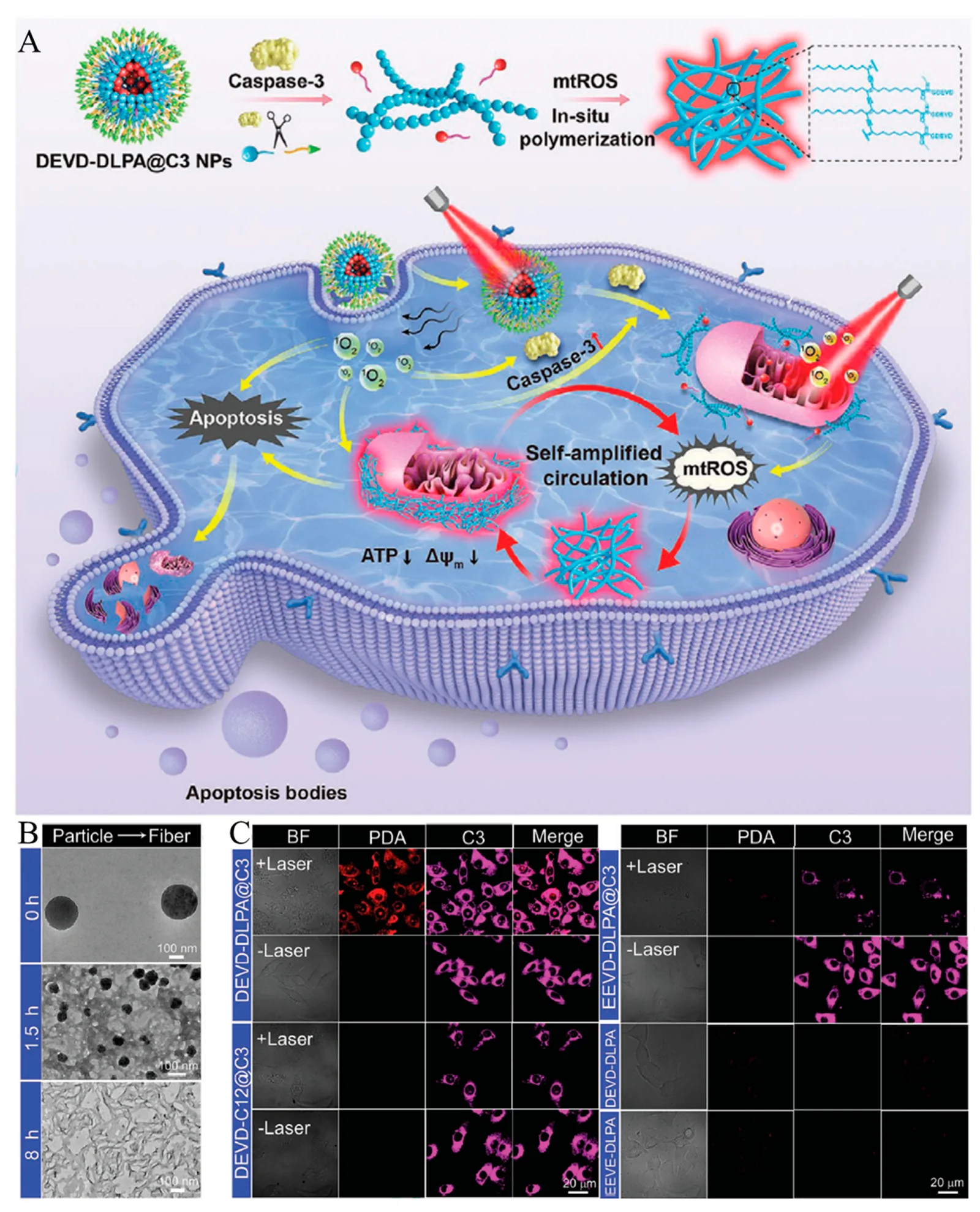
Caspase-3-triggered morphological transformation and intracellular polymerization of DEVD-DLPA@C3 NPs. (**A**) Schematic illustration of the caspase-3-triggered NP-to-nanofiber transformation, mtROS-mediated in situ polymerization, and self-amplified mitochondrial oxidative damage. Initial photodynamic treatment activates caspase-3, which cleaves the DEVD sequence and induces NP disassembly and nanofiber formation. Subsequent mtROS production promotes diacetylene polymerization, resulting in dense polymerized nanofibers and amplified mitochondrial dysfunction. (**B**) Time-dependent TEM images showing the transformation from spherical NPs to intermediate fibrous structures and an elongated nanofibrous network following incubation with caspase-3. (**C**) Intracellular fluorescence images showing PDA formation after two cycles of 655 nm laser irradiation. Red, polymerized diacetylene (PDA); magenta, C3 fluorescence; BF, bright field. Adapted with permission from Ref. [60]. Copyright © 2022 Wiley-VCH GmbH.

Ma et al. developed the caspase-3-responsive NP diacetylene-containing lipidated peptide amphiphile (DEVD-DLPA@C3) to amplify PDT by continuously inducing shape conversion and covalent polymerization of nanostructures within the cells (Figure 3A) [60]. The nanocarrier DEVD-DLPA consists of a GDEVDRRRRGDS sequence and an N-terminal diacetylene lipid, and the RRRRGDS site binds integrin to target cancer cells. Here, the mitochondria-targeting cyanine photosensitizer C3 containing triphenylphosphonium (TPP) was loaded at a peptide-to-C3 molar ratio of 3:1. C3 encapsulation efficiency and drug loading were 80.3% and 11.1%, respectively, and the formed spherical DEVD-DLPA@C3 NP showed a size of 196 ± 5 nm in TEM and 210 ± 15 nm in DLS. However, the free-C3 and non-polymerizable or cleavage-insensitive controls differed in carrier architecture or responsiveness; therefore, the enhanced outcome supports the contribution of transformation and polymerization without isolating either as the sole determinant.

This transformation begins with the sequential actions of exogenous light, endogenous caspase-3, and ROS. The first 655 nm laser irradiation activates C3 to generate singlet oxygen (^1^O_2_) and ROS, induces mitochondrial dysfunction and the intrinsic apoptosis pathway, and activates caspase-3. Caspase-3 cleaves the aspartic acid–glutamic acid–valine–aspartic acid (DEVD) sequence of DEVD-DLPA, releasing the RRRRGDS fragment and C3 and changing the molecular packing parameter of the remaining diacetylene-GDEVD fragment. The critical packing parameters of DEVD-DLPA and diacetylene-GDEVD were calculated to be 0.1228 and 0.3823, respectively, and these structural changes led to the disassembly of spherical NPs and one-dimensional nanofibers.

Before Caspase-3 treatment, spherical NPs of approximately 200 nm were observed. However, after 1.5 h, particles and fibers appeared together, and after 8 h, most of them were converted into an elongated nanofibrous network (Figure 3B). Subsequently, ROS generated in the mitochondria induced topochemical polymerization of diacetylene groups aligned inside the nanofiber, forming a covalent polydiacetylene (PDA) nanofiber. In simple DEVD-DLPA NPs, the arrangement of the diacetylene group was not suitable for polymerization. Therefore, effective polymerization did not occur with ultraviolet irradiation, laser irradiation, pH change, or caspase-3 treatment alone. In other words, caspase-3-mediated cleavage first creates a molecular alignment suitable for nanofiber formation and then ROS polymerizes the aligned diacetylene monomer.

This sequential intracellular morphological transformation occurs within the tumor cells, and the final structure is predominantly localized at the mitochondrial membrane. DEVD-DLPA@C3 was mostly internalized within approximately 4 h of incubation with 4T1 breast cancer cells. After the first laser irradiation and an additional 8 h of incubation, the colocalization coefficient between C3 and mitochondria increased from 71% to 86% because C3 released following caspase-3-mediated cleavage accumulated at the mitochondrial membrane through its TPP group. The high ROS levels generated after the second laser irradiation promoted the intracellular polymerization of the diacetylene-containing nanofibers. Accordingly, strong PDA-specific red fluorescence was observed in the laser-irradiated DEVD-DLPA@C3 group, whereas substantially weaker or negligible PDA fluorescence was detected in the non-irradiated and nonpolymerizable control groups (Figure 3C). Supplementary colocalization analysis further indicated that the polymerized nanofibers were predominantly localized in the mitochondrial membrane. Therefore, morphological transformation enhanced mitochondrial localization, whereas subsequent polymerization stabilized the retention of C3 and the nanofibrous structures in the organelle.

C3 and polymerized nanofibers accumulated in the mitochondria induced a decrease in the mitochondrial membrane potential, ATP depletion, and a mitochondrial ROS (mtROS) burst. The increased mtROS levels promote additional caspase-3 activation and diacetylene polymerization, forming a positive-feedback cycle, leading to ROS–caspase-3–nanofiber formation–polymerization–mtROS. In the DEVD-DLPA@C3 treated group, cleaved caspase-3 expression increased approximately 8.5 times compared to the untreated control. After two rounds of laser irradiation, the viability of 4T1 cells decreased to 17.4% and the apoptotic cell rate reached 90.1%. This was higher than the apoptosis rates of 35%, 54%, and 83% observed in the C3, non-polymerizable DEVD-C12@C3, and caspase-3-insensitive EEVE-DLPA@C3 treatment groups, respectively. Additionally, cancer cell migration and invasion were significantly inhibited in wound healing and transwell assays.

In subcutaneous 4T1 breast tumor-bearing mice, DEVD-DLPA@C3 accumulated in tumors after intravenous injection and showed 94.1% tumor growth inhibition when irradiated with a 655 nm laser twice at 8-h intervals. Hematoxylin and eosin and terminal deoxynucleotidyl transferase dUTP nick-end labeling (TUNEL) analyses of tumor tissues also confirmed the most extensive tissue damage and apoptosis. Body weight changes and pathological damage to major organs were not evident. Taken together, this study demonstrates a small-to-large morphological transformation, in which a spherical NP of approximately 210 nm is converted into a mitochondrial nanofiber by intracellular caspase-3, and the structure is subsequently stabilized through mtROS-mediated polymerization. This sequential nanofiber formation and polymerization formed a positive-feedback cycle, leading to ROS–caspase-3–nanofiber formation–polymerization–mtROS, which self-amplified PDT. The studies discussed in this section are summarized in Table 2. The group size for the cited tumor growth efficacy values was not explicitly identified in the main article. The primary study examined organ fluorescence at 24 h and major organ histology after treatment, but did not directly determine the degradation products, excretion routes, or persistence of the caspase-3-triggered polymerized nanofibers beyond the efficacy window.

For gold aggregates, covalently cross-linked microdepots, polymerized peptide fibers, short-term body weight, serum chemistry, and major organ histology did not establish degradation or clearance. Most of the small-to-large and multistage studies reviewed did not directly quantify the long-term disassembly products, excretion routes, or structural persistence beyond the efficacy window. P-CyPt is a notable exception because its primary study quantified the early urinary and fecal elimination of molecular and platinum-containing species over 0–12 h. For the other reviewed platforms, the available short-term body weight, blood, histology, fluorescence, efficacy, and survival measurements did not establish complete degradation, excretion, or long-term structural clearance. This recurrent absence should be interpreted as a systematic translational gap rather than as evidence of long-term safety.

## 4. Multistage, Sequential Assembly–Disassembly, and Reassembly

Unidirectional size changes, such as large to small or small to large transformations, can address specific delivery barriers, but cannot satisfy all conflicting requirements across the entire delivery process, from systemic circulation to intracellular drug release [34,35,50]. To solve this problem, multistage transformation systems are being developed, in which assembly, disassembly, and reassembly occur sequentially in different biological compartments [50]. In these systems, each intermediate structural state performs a distinct delivery function, such as maintaining circulatory stability, enhancing tumor penetration, promoting local retention or cellular uptake, and enabling intracellular drug release [50]. Therefore, the final treatment performance was determined by the order and spatial location of the structural transitions rather than by a single initial or final size [34,50].

Multistage transformations can be implemented using various paths [80]. After penetrating the tumor tissue, a molecular precursor may assemble into NPs at the tumor cell membrane and subsequently disassemble under intracellular reducing conditions to release the drug [65]. Conversely, large core-satellite assemblies may release small penetrating NPs into the TME, whereas the exposed components reassemble into nanogels that form local reservoirs [66]. It is also possible to disassemble the initial NPs and then reassemble them into smaller NPs [81]. Thus, disassembly does not necessarily terminate carrier function but may serve as an intermediate step that generates a subsequent structure with a distinct delivery function [65,66,81].

An important limitation of the current literature is that local stimulus levels are rarely quantitatively or spatially correlated with the degree of structural transformation. Therefore, spatially heterogeneous pH, MMP activity, hypoxia, and redox conditions may produce regional differences in the extent and kinetics of transformations, potentially resulting in partial or asynchronous transitions [82,83,84,85,86,87,88,89]. This limitation becomes particularly important as the number of transformation steps increases, because the design burden of ensuring that each transition occurs at the appropriate location and time also increases [50,80]. If the first conversion occurs too rapidly, enlarged structures may become trapped near vessels before reaching deeper tumor regions; if it occurs too slowly, the system may be cleared from the tumor before assembly or disassembly can proceed [50]. Similarly, if the upstream conversion is incomplete because the local stimulus does not reach the required activation threshold, subsequent reactions may become chain-limited. Therefore, the intermediate structure, activation threshold, and conversion rate must be characterized [50].

This section compares representative systems in which the molecule-to-NP assembly, NP disassembly, nanogel or nanofiber reassembly, and intracellular drug release occur sequentially. Accordingly, the advantages of multistage transformations are evaluated not by the number of reaction steps alone, but by the functional correspondence between each structural state and the biological barrier it is intended to overcome.

### 4.1. Enlargement/Assembly-to-Disassembly Trajectories

Chen et al. developed the GBI-10 aptamer-coated crosslinked dendri-graft poly-L-lysine (DGL) NP s(DGL)n@Apt, a multistage shrinkage NP that releases ultrasmall particles after transient enlargement in response to a hypoxic concentration gradient, to treat pancreatic ductal adenocarcinoma (PDAC), where deep tumor penetration of drugs is limited by the dense matrix [49]. This system is a structure in which DGL is cross-linked with a hypoxia-responsive linker, gemcitabine monophosphate (pGem), and HJC0152, a signal transducer and activator of transcription 3 (STAT3) inhibitor, were loaded together, and the surface was coated with the GBI-10 aptamer, which recognizes tenascin-C overexpressed in the ECM of PDAC. The negatively charged particles, approximately 104 nm in size, shield the positively charged DGL core, improving its stability in circulation and tumor accumulation. The pGem loading was 2.1 ± 0.4%, and the therapeutic groups received the same pGem dose of 10 mg/kg. The enhanced penetration, retention, and therapeutic response supported the meaningful contribution of the multistage size transformation, whereas the overall efficacy reflected its integrated action with aptamer targeting and co-delivered HJC0152.

Upon reaching the tumor, the GBI-10 aptamer binds to tenascin-C and dissociates from the particle surface, exposing a positively charged internal structure. In a weak hypoxic environment, partial reduction in the hypoxic-responsive linker temporarily enlarges the dense NPs of approximately 100 nm into a loose structure of approximately 1 μm, increasing their retention in the tumor. Subsequently, in a more severely hypoxic environment, the linker is further decomposed, the cross-linked structure is dismantled, and pGem-loaded DGL particles smaller than 10 nm are released. Therefore, this system corresponds to a large-to-small transformation, in which particles of approximately 104 nm are functionally converted into delivery units of less than 10 nm. However, hypoxia-gradient-driven multistage shrinkage is distinguished from simple shrinkage in that size reduction does not occur through direct contraction of the entire particle but is realized through transient enlargement and disassembly mediated by ultrasmall-particle release.

The released ultra-small DGL particles passed through the dense PDAC matrix and penetrated deep into the tumor spheroids, after which they were internalized by cancer cells via clathrin-mediated endocytosis and macropinocytosis. This delivery method improved the intracellular delivery of pGem by bypassing the low expression of nucleoside carriers required for cellular uptake of gemcitabine. Simultaneously released HJC0152 suppressed activated STAT3 signaling in tumor cells and pancreatic stellate cells and reduced secretion of TGF-β1, alleviating collagen-rich stroma. Accordingly, the penetration of ultra-small particles and the anticancer effect of pGem were further improved, and apoptosis by pGem and autophagy by DGL were also induced.

In the orthotopic Pan02 PDAC model, s(DGL)n@Apt inhibited tumor growth, liver and peritoneal metastases, and prolonged survival. It also reduced the proportion of regulatory T cells while activating the antitumor immune response by increasing the tumor infiltration of CD8^+^ cytotoxic T cells, CD4^+^ helper T cells, and mature dendritic cells within tumor-draining lymph nodes. In summary, in this study, transient enlargement was an auxiliary step to increase intratumoral retention, and deep tumor penetration of sub-10 nm DGL particles generated by subsequent disassembly was the main size-transformable delivery function. Accordingly, the system of Chen et al. can be classified as a case of shrinkage for deep tumor penetration, and the detailed path can be defined as transient enlargement-disassembly mediated multistage shrinkage. This sequential transformation links tumor retention by transient enlargement and deep tumor penetration by ultrasmall particle release, demonstrating a multistage shrinkage strategy that integrates pGem delivery, STAT3 inhibition, and stromal remodeling. The efficacy experiment used eight mice per group at an equal pGem dose of 10 mg/kg, and selected immune measurements used three mice per group. The primary study reported therapeutic, immune, and survival outcomes for the integrated multistage system but did not directly quantify the long-term degradation, excretion, or structural persistence of the transient assemblies or released DGL NPs.

Wen et al. developed a cisplatin prodrug that underwent sequential self-assembly and disassembly in tumors to address the trade-off between deep penetration of small molecules and greater intratumoral retention and cellular uptake of larger nanostructures [65]. The developed P-CyPt was designed to disperse at the molecular level when administered, quickly spread into the tumor tissue, and assemble into NPs on the surface of the tumor cells to increase local retention. The NPs were internalized and disassembled to release cisplatin.

**Figure 4:**
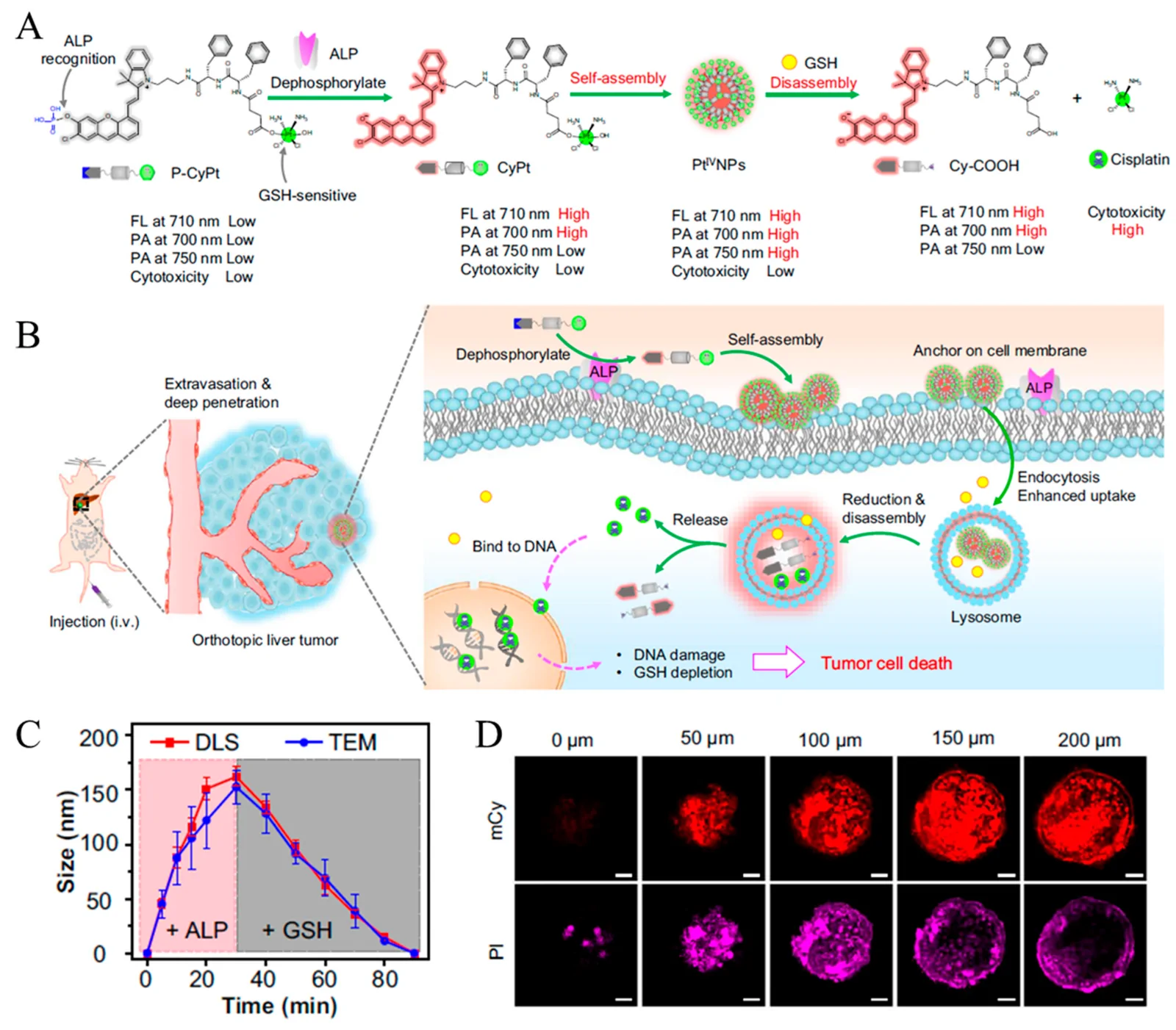
Sequential ALP-triggered self-assembly and GSH-mediated disassembly of P-CyPt for tumor theranostics. (**A**) Chemical transformation of P-CyPt through ALP-mediated dephosphorylation and self-assembly into Pt(IV) NPs, followed by GSH-induced disassembly and cisplatin release. (**B**) Proposed mechanism involving tumor extravasation and deep penetration of P-CyPt, ALP-triggered self-assembly at the tumor-cell membrane, cellular internalization, and intracellular GSH-mediated disassembly and drug release. (**C**) Time-dependent changes in particle size measured by dynamic light scattering (DLS) and transmission electron microscopy (TEM), showing NP formation following ALP treatment and subsequent disassembly after GSH addition. (**D**) Fluorescence images of P-CyPt-derived mCy and propidium iodide staining at different depths of HeLa multicellular tumor spheroids, demonstrating deep spheroid penetration and associated cell death. Adapted from Ref. [65] under the CC BY 4.0 license. Copyright © 2023 The author(s).

P-CyPt consists of a near-infrared merocyanine fluorophore (mCy), a phosphate group for alkaline phosphatase (ALP) recognition, a reduction-responsive platinum(IV) (PtIV) cisplatin prodrug, and a hydrophobic D-phenylalanine-D-phenylalanine dipeptide that promotes self-assembly (Figure 4A). The initial P-CyPt maintained a hydrophilic molecular dispersion state and exhibited a low fluorescence signal, low photoacoustic (PA) signal, and low cytotoxicity. This small molecule state is advantageous for extravasation and diffusion within tumor tissue, but has the limitation that it is difficult for it to remain in tumor tissue for a long period of time. As P-CyPt is a covalent molecular prodrug, conventional encapsulation-based loading parity is not applicable. Therefore, comparisons with cisplatin and preformed Pt(IV) NPs evaluate different chemical and structural presentations; they support the utility of sequential design but do not isolate structural transformation as the only causal variable.

When P-CyPt reaches the ALP expressed on the tumor cell membrane, the phosphate group is removed and converted to CyPt. In this process, as the partition coefficient increases from 0.68 to 2.50 and the hydrophobicity of the molecule increases, spherical Pt(IV) NPs with an average size of approximately 160 nm are assembled through π–π stacking, van der Waals interaction, and hydrogen bonding. In other words, this step is not a simple enlargement of preformed NPs, but a molecule-to-NP transformation in which the small-molecule prodrug that has reached the tumor self-assembles extracellularly. The resulting Pt(IV) NPs were anchored to the ALP-expressing tumor cell membrane, thereby reducing back-diffusion and increasing local retention and cellular uptake (Figure 4B).

Pt(IV) NPs internalized by cells undergo a second structural transition because of the high concentrations of glutathione (GSH). GSH reduces platinum(IV) to platinum(II), generating negatively charged Cy-COOH and cisplatin (CDDP), thereby inducing NP disassembly. Following ALP treatment, the particle size increased to approximately 160 nm as Pt(IV) NPs were formed and subsequently decreased following GSH addition, as confirmed by DLS and TEM (Figure 4C). In the presence of 10 mM GSH, CyPt was completely converted to Cy-COOH within 60 min, resulting in NP disassembly and cisplatin release. This process rapidly releases cisplatin from inside the cell and simultaneously depletes intracellular GSH by approximately 37.3%, thereby inducing both DNA damage and disturbances in redox homeostasis.

Stepwise conversion of P-CyPt provided better penetration and cytotoxicity than fixed-size preformed Pt(IV) NPs. In HeLa multicellular tumor spheroids, molecularly dispersed P-CyPt penetrated deeply into the spheroids and induced cell death in the inner regions following in situ NP formation and intracellular disassembly (Figure 4D). In HeLa cells, the apoptotic cell population after P-CyPt treatment was approximately 64.5%, which was higher than that after treatment with CDDP (approximately 5.20%) or the preformed Pt(IV) NPs (approximately 7.33%). In addition, the half-maximal inhibitory concentration (IC_50_) of P-CyPt against HeLa cells was 6.76 ± 0.68 μM, compared with 14.47 ± 1.60 μM for CDDP, whereas P-CyPt showed relatively low toxicity toward normal human embryonic kidney HEK-293T cells.

In the subcutaneous HeLa tumor model, the tumor accumulation of P-CyPt reached approximately 20.3% of the injected dose per gram of tissue (%ID/g) at 4 h, exceeding the corresponding values for preformed Pt(IV) NPs (approximately 4.7%ID/g) and CDDP (approximately 3.6%ID/g). In the P-CyPt treatment group, no apparent tumor growth was observed over 21 days, and P-CyPt did not induce the increases in blood urea nitrogen and creatinine levels observed in the CDDP-treated group. Strong tumor inhibition and accurate tumor imaging were confirmed in an orthotopic luciferase-expressing HepG2 liver tumor model. In this system, the same prodrug facilitated tumor penetration in its molecularly dispersed state, enhanced local retention and cellular uptake after NP assembly, and enabled intracellular drug release after disassembly. This study demonstrates that beyond the direction of size transformation, the biological location and temporal sequence of each structural transition determine its delivery function and ultimate therapeutic outcome. The subcutaneous HeLa efficacy experiment used n = 4 independent animals per group. This study examined pharmacokinetics, metabolism, and clearance: P-CyPt had a blood half-life of 0.54 ± 0.06 h, and P-CyPt, Cy-COOH, and platinum-containing species were measured in urine and feces collected over 0–12 h, supporting combined renal and hepatobiliary excretion. These data provide evidence of early clearance, although they do not establish complete mass balance or long-term tissue clearance after repeated dosing.

### 4.2. Disassembly-to-Reassembly Trajectories

Dosta et al. developed a dual-sensitive NP system that disassembles in the TME and then reassembles into a nanogel to resolve the conflicting demands that a locally administered drug carrier stay long enough in glioblastoma (GBM) and spread deeply into the invasive tumor tissue [66]. This platform is initially administered as relatively large NP assemblies but dissociates into component NPs in response to MMP-9 and acidic pH. The reactive functional groups exposed in this process form a nanogel at the tumor site, which acts as a local reservoir that continuously releases small drug-loaded NPs.

Dual-NPs were designed as core satellite structures in which DOX-conjugated dendrimer NPs of approximately 14.5 nm were assembled around oxidized dextran NPs of approximately 152–162 nm. The two NPs were connected through copper-free click chemistry between dibenzocyclooctyne and azide, and the final dual-NP exhibited a hydrodynamic diameter of approximately 169 nm and a zeta potential of +13.7 mV. DOX was conjugated to each dendrimer through an acid-cleavable aconityl linker, and an average of approximately seven DOX molecules were conjugated to each dendrimer NP.

The dual-sensitive linker connecting the two NP components included an MMP-9-sensitive VPLSLYSG peptide sequence and a pH-sensitive imine bond. MMP-9 in the GBM microenvironment cleaves the peptide linker to release dendrimer NPs, and the acidic pH hydrolyzes the imine bond on the surface of dextran NPs. When MMP-9 was present, approximately 60% of the linker was cleaved within 8 h and approximately 80% within 48 h, and the imine linker was also cleaved faster at pH 6.8 than at pH 7.4. These two reactions exposed the amine groups on the dendrimer NPs and the aldehyde groups on the dextran NPs, leading to subsequent structural transitions.

The exposed amine and aldehyde groups form new cross-links through a Schiff base reaction, resulting in their reassembly into a porous nanogel at the tumor site. In vitro, nanogel formation was completed within approximately 21.8 ± 1.8 s following MMP-9 treatment, whereas no gel was formed in the absence of MMP-9. The resulting nanogel served as a tumor-bound reservoir, releasing small DOX-conjugated dendrimer NPs over time. Thus, the same disassembly process releases small penetrable carriers while enabling the remaining components to reassemble into a persistent local reservoir. DOX was covalently associated with the dendrimer component, rather than being compared solely through conventional encapsulation loading. Enhanced penetration and retention support the meaningful contribution of disassembly and reassembly, although linker cleavage, charge exposure, and nanogel formation also contribute to the overall therapeutic response.

In GL261 glioblastoma spheroids, dual-NPs treated with MMP-9 showed greater penetration into the middle and core regions than the untreated dual-NPs. As the small dendrimer NPs released from the nanogel moved to the center of the spheroid over time, the fluorescence signal in the inner region increased on day 5. The half-maximal inhibitory concentration (IC_50_) of MMP-9-treated dual-NPs was 1.22 μM, compared with 4.99 μM for untreated dual-NPs. Additionally, spheroid viability on day 5 was approximately twofold lower in the MMP-9-treated group, indicating that the release and penetration of small dendrimer NPs due to structural transformation led to improved cytotoxicity.

In the orthotopic GL261-luciferase glioblastoma model, dual-NPs were detected at the tumor site for up to 6 days after a single intratumoral injection. On day 2, the retention was approximately twice as high as that of free dendrimer NPs, and even on day 6, approximately 40% of the initial dual-NPs remained, whereas less than 10% of the free dendrimer NPs remained. The median survival of the dual-NP treatment group was 30 days, which was longer than that of the dendrimer NP group (22 days), free doxorubicin group (21 days), and untreated group (18 days). In 25% of the treated animals, no residual tumors were observed, even after 2 months. This study showed that disassembly of the initial assembly simultaneously induces the release of small penetrating NPs and reassembly of the local nanogel, thereby enabling the conflicting delivery functions of tumor retention and deep tumor penetration to be spatially and temporally separated. Retention measurements were performed using n = 5, and survival analysis was performed using n = 6–12 biologically independent animals. The primary study demonstrated local structural persistence for up to six days and followed survival for as long as two months, but did not directly quantify chemical degradation products, systemic excretion, or clearance of the intracranial nanogel and its components after the local reservoir dissipated.

**Figure 5:**
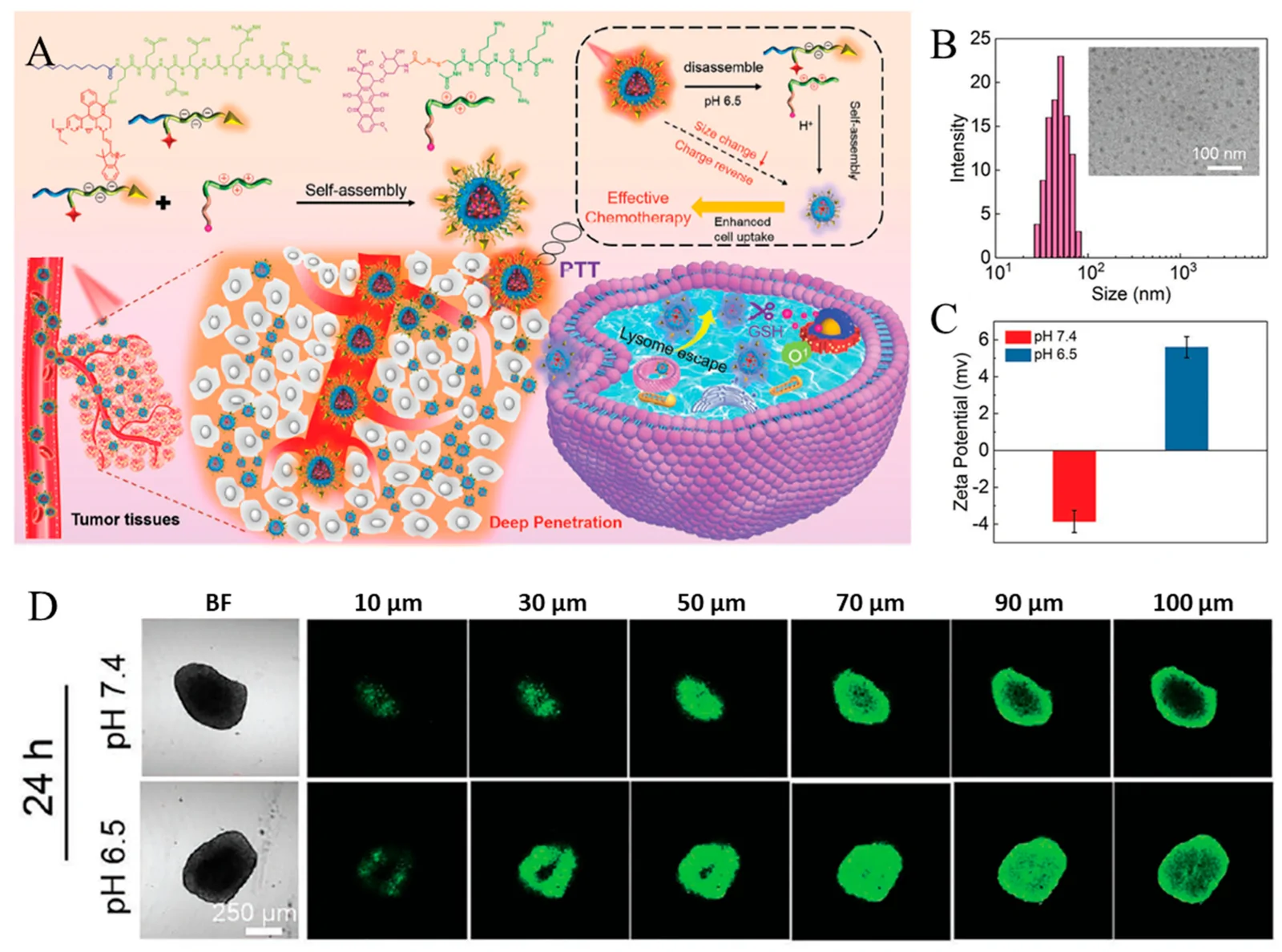
Tumor-responsive size transformation and enhanced penetration of PP NPs. (**A**) Schematic illustration of PP NP preparation and their pH- and NIR-responsive size reduction, charge reversal, deep tumor penetration, cellular uptake, and combined photo–chemotherapy. (**B**) Size distribution and TEM image of PP NPs after incubation at pH 6.5 and NIR laser irradiation. (**C**) Zeta potentials of PP NPs at pH 7.4 and 6.5. (**D**) Penetration of PP NPs into multicellular tumor spheroids at pH 7.4 and 6.5 over 24 h. Adapted with permission from Ref. [81]. Copyright © 2021 Wiley-VCH GmbH.

Ma et al. developed a bicomponent peptide NP that was disassembled by stimulation and then reassembled into smaller NPs to combine the circulatory stability and tumor accumulation of large NPs with deep tumor penetration and cellular uptake of small NPs (Figure 5A) [81]. This system is maintained as PP NPs of approximately 170 nm under physiological conditions; however, in response to the acidic microenvironment of the tumor and NIR irradiation, the existing assembly is disassembled and reassembled into smaller NPs of approximately 15–20 nm.

PP NPs were prepared by mixing PA1, a negatively charged peptide amphiphile, and PA2, a positively charged peptide prodrug, in a 1:1 molar ratio. PA1 consists of lauric acid, an NIR dye, three glutamic acid residues, and an integrin-targeting RGDS sequence responsible for hydrophobic self-assembly, phototherapy, negative-charge formation, and tumor targeting. PA2 is a positively charged prodrug that links doxorubicin to a lysine-rich peptide through a GSH-cleavable disulfide bond. The two components formed spherical NPs of approximately 170 nm through electrostatic attraction, hydrophobic interactions between the NIR dye doxorubicin and the alkyl chain, and π–π stacking.

At physiological pH 7.4, PP NPs had a size of approximately 170 nm and a near-neutral surface charge of −3.9 mV and remained relatively stable for 7 days even in conditions containing serum and protease. This initial structure was designed to reduce non-specific interactions in the blood while taking advantage of the tumor accumulation of relatively large NPs. In pharmacokinetic analysis, distribution half-life and elimination half-life were 1.65 ± 0.21 h and 22.35 ± 2.67 h, respectively. After intravenous injection, the fluorescence signal at the tumor site peaked at approximately 12 h and was maintained at 24 h, indicating that the initial large assembly contributed to long-term circulation and tumor delivery. The therapeutic component was incorporated as a peptide prodrug, making conventional drug-loading parity less informative than dose and molecular composition matching. The enhanced penetration and therapeutic response support a meaningful contribution to size transformation, whereas acidity-induced disassembly, reassembly, charge exposure, drug activation, and NIR action operate as an integrated system.

In the acidic TME, the electrostatic balance between PA1 and PA2 is disturbed due to changes in the protonation of the peptide component, making the initial assembly of approximately 170 nm unstable. Due to this change, the large PP NPs were disassembled, and PA1 and PA2 were interpreted to be reassembled into small NPs of approximately 15–20 nm based on the remaining hydrophobic interactions and π–π stacking (Figure 5B). Because small particles were observed after 8 h at acidic pH alone, pH is the main stimulus for structural conversion, and the photothermal effect generated by 730 nm NIR irradiation can be viewed as an auxiliary stimulus that promotes molecular rearrangement and reassembly. During the reassembly process, the zeta potential also changed from −3.9 mV to +5.6 mV, and the small size and increased positive charge contributed to tumor penetration and improved cellular uptake, respectively (Figure 5C).

In the 4T1 multicellular tumor spheroid model, fluorescence from PP NPs was detectable to a depth of approximately 50 μm at pH 7.4 and approximately 110 μm at pH 6.5 after 12 h. The 24-h images further confirmed the enhanced spheroid penetration and broader distribution of the transformed NPs under acidic conditions (Figure 5D). In the subcutaneous 4T1 tumor model, NP-associated fluorescence was distributed not only to the periphery of the tumor but also to the center, showing a penetration depth of approximately 600 μm or more. After entering the cell, intracellular glutathione cleaves the disulfide bond of PA2, releasing doxorubicin. Under pH 6.5 and 50 μM glutathione conditions, cumulative DOX release reached 85.4 ± 6.1%, while under neutral conditions it remained at approximately 17.4%.

The reassembled small NPs maintained the aggregated state of the NIR dye and exhibited photothermal activity and singlet oxygen generation abilities similar to those of the initial large NPs. When 730 nm irradiation and PP NPs were used together, 4T1 cell viability decreased to approximately 20.2%, and the apoptotic cell population reached approximately 70%. In vivo, the strongest inhibition of tumor growth was observed in the PP NPs plus laser group. This study shows that the disassembly of NPs is not a terminal step, indicating a loss of carrier function, but can be used as an intermediate step for reassembly into new nanostructures. In other words, the stability in circulation and tumor accumulation functions of large NPs are converted to deep tumor penetration and cellular uptake functions of reassembled small NPs, while enabling phototherapy and chemotherapy. In this system, disassembly and small NP reassembly induced by the acidic TME convert the initial structure favorable for tumor accumulation into a delivery state suitable for deep tumor penetration and cellular uptake. Subsequently, the antitumor effect was strengthened by linking the drug release and photothermal/photodynamic activity through NIR irradiation. Therefore, this study demonstrates a size-transformable delivery strategy that sequentially connects tumor accumulation, deep tumor penetration, and multimodal therapy using disassembly–reassembly. The studies discussed in this section are summarized in Table 3. The primary study assessed major organ histology at 24 h and hematology and serum biochemistry on day 16, but did not directly quantify the degradation products, excretion, or long-term persistence of the reassembled peptide NPs.

## 5. Conclusions

Size-transformable NPs temporally and spatially assign a structural state suitable for each biological barrier, instead of applying a fixed size to all delivery steps. According to the function-based classification in this review, large-to-small transformations are mainly responsible for deep tumor penetration and cellular uptake after tumor accumulation, whereas small-to-large transformations are responsible for tumor and intracellular retention, drug depot formation, and organelle damage. Multistage systems sequentially distribute the accumulation, penetration, retention, and release functions into different intermediate structures. Therefore, the key conclusion of this review is that function cannot be predicted solely by the direction of size change and that the location and sequence of transitions, together with the reported temporal behavior where available, as well as the accompanying charge, surface, shape, stiffness, and corona changes, determine therapeutic function.

However, the direction of the size change alone is not sufficient to predict system function or explain treatment effects. The same size reduction can increase deep tumor penetration if it occurs in the tumor extracellular space. However, if it occurs in the lysosome, drug release or fluorescence activation is the main function. Similarly, extracellular aggregation may inhibit intratumoral backspreading and increase local retention, whereas intracellular or organelle-localized assembly may contribute to the inhibition of exocytosis, organelle damage, or activation of specific apoptotic pathways. Therefore, a size-transformable system must be evaluated considering not only the initial and final size, but also the transformation stimulus, biological site, intermediate structure, transformation kinetics, surface charge and shape changes, and biological function responsible for each change. Across the reviewed studies, nominally matched cargo doses were more common than the complete matching of loading, baseline charge, ligand density, and surface chemistry. Accordingly, study-level efficacy differences were interpreted as evidence for the contribution of structural transformation only when supported by an appropriate non-transformable control and not as proof that size change was the sole causal variable.

An important limitation of the current literature is that there is insufficient direct evidence that the intended size changes occur in vivo. Many studies have inferred transformation by linking the results of in vitro DLS or electron microscopy with the increase in tumor distribution after treatment. However, it is difficult to clearly distinguish between particle structural changes and drug release based solely on the increase in the fluorescence signal. In future research, it will be necessary to directly verify the state before and after the change and the biological site by combining Förster resonance energy transfer, environment-sensitive probes, photoacoustic (PA) spectral changes, intravital microscopy, cryogenic electron microscopy, and structural analysis of the recovered tumor tissue. In non-spherical aggregates and nanofibers, it is difficult to interpret the hydrodynamic diameter obtained by DLS as the actual structure size; therefore, multiple analysis methods appropriate for the shape are also required.

The comparative efficacy must also be interpreted in the context of drug dose, loading, baseline charge, ligand density, and irradiation conditions. When the primary study reported matched drug doses and otherwise comparable treatment conditions, it was noted in the study-specific discussion. When the surface chemistry or loading differed, the therapeutic improvement was not exclusively attributed to the size transformation. Verified endpoint-specific sample sizes were retained where explicitly available in the primary figures or legends, and not reported (NR) was used when the relevant n values could not be identified. No new statistical values were inferred from the incomplete summary data.

Temporal compatibility is important, but most primary studies have not reported formal transformation t50 values, rate constants, or concurrent in vivo measurements of the structural state and tumor residence. The reported observations range from seconds to days and often represent different endpoints, including linker cleavage, gel formation, particle size measurement, drug release, and biodistribution. These values could not be treated as directly comparable kinetic constants. Multiday in vitro observations may reflect the gradual transformation of a tumor-retained fraction rather than a transformation of the circulating population. Future studies should concurrently measure the structural state, stimulus level, tissue residence, penetration, uptake, and release under physiologically relevant serum and matrix conditions.

Protein corona formation represents an additional source of discrepancy between transformation measured in simplified media and that observed in vivo. Protein adsorption can alter apparent hydrodynamic size, mask cleavable linkers or targeting ligands, stabilize or destabilize colloidal assemblies, and alter enzyme access to responsive interfaces. Consequently, transformation measured in buffer at a fixed pH, enzyme, or glutathione concentration may not reproduce the rate or extent observed in the plasma, tumor interstitium, or intracellular compartments. Corona composition may also modify the circulation half-life, organ distribution, cellular uptake, and clearance both before and after transformation [90]. Direct comparisons of pharmacokinetics before and after structural transformations were uncommon among the reviewed studies, representing a major gap in the evidence. Therefore, future studies should characterize the composition, transformation kinetics, and pharmacokinetic behavior of the corona under physiologically relevant conditions.

However, safety and clinical manufacturing remain significant challenges. Aggregates, inorganic NPs, and covalently polymerized nanofibers that increase tumor retention may be advantageous for short-term therapeutic effects; however, long-term degradation and clearance from the body must be fully verified. Complex multicomponent systems can increase batch-to-batch variations in drug loading, linker density, initial particle size, and stimulus responsiveness, and reduced efficiency in one of several sequential reactions can limit the overall delivery process. Therefore, clear functional transitions should be implemented with as few components as possible, and mass production, sterilization, storage stability, and quality control standards should be considered during the early development stages. In addition, it is important to continuously analyze the structure before and after the change, location and time of the transition, distribution within the tumor, and subsequent therapeutic function to confirm the role of the complex structural transition in the overall delivery process.

To make the biomarker-guided design more concrete, Table 4 summarizes the reported microenvironmental features across 12 commonly used tumor models, including the models represented in this review and additional benchmark models. As extracellular acidity, protease activity, and hypoxia depend strongly on the implantation site, tumor size, disease stage, sampling region, and assay method, this table emphasizes the reproducibly reported model characteristics rather than combining heterogeneous measurements into universal numerical ranges. Therefore, the model-specific feature map in Table 4 should be used to select candidate stimuli and validation assays and not as a set of transferable activation thresholds.

Future developments in size-transformable NPs should focus on forming the structures required for clinically important barriers at the exact location and time rather than implementing larger changes or more reaction steps. Therefore, the structure before and after the change must be directly confirmed in vivo, and the process by which the size change is linked to the surface charge change, drug release, and phototherapy to form a sequential delivery function must be identified. By combining transformation monitoring with biomarker-guided design, which reports model- and compartment-specific ranges for vascular structure, extracellular matrix density, enzyme activity, and immune microenvironment, it is possible to select patients with a high likelihood of response and optimize treatment conditions. Ultimately, size transformation is not an end in itself but a means of readjusting the mobility, retention, cellular accessibility, and therapeutic action of nanocarriers to suit the delivery route and should become the central design principle for clinical translation in this field.

## Figures and Tables

**Table 2:** Particle aggregation/drug-depot and shape/morphology-transforming platforms.

Platform/Type	Size/Structural Trajectory	Trigger/Site	Delivery Function/Cargo/Modality	Model	Reported Time/In Vivo Evidence	Ref.
AuNP aggregate (metal NP)	38.2 nm → 263.2 nm aggregates; Covalent aggregation	Furin/ tumor ECM	Tumor and intracellular retention; DOX + HCQ; Key animal comparisons n = 5	MCF-7/ADR	NR; Evidence: None	[78]
D-NP/C-NP (polymer NPs)	~65 nm NPs → ~2.6 μm depot; Cys–CBT condensation	Acidic pH/extracellular TME	Local drug-depot formation and tumor retention; BB94; DOX + NLG919 + BLZ945; Major efficacy/immune analyses n = 5	4T1	aggregates 2–24 h in vitro; observed at 6 h in vivo; Evidence: Direct	[57]
AuNPs-P-DA (metal NP)	50.4 nm → 420.6 nm aggregates; Charge change; aggregation	Acidic pH/tumor milieu	Tumor retention and radiosensitization; DOX + RT; Colony assay n = 3; apoptosis n NR	KYSE-510	420.6 nm at 4 h, pH 6.5 in vitro; Evidence: Indirect	[55]
AGLR (peptide NP)	10–15 nm NPs → nanofibers; Cleavage; reassembly	MMP-2/tumor ECM	Initial penetration followed by prolonged retention; DOX; Endpoint-specific efficacy n NR	H22	fiber emergence 12 h; predominant by 72 h with MMP-2, in vitro; Evidence: Indirect	[79]
NP-NH-D5 (peptide NP)	~60 nm NP → ~12 nm-diameter fibers; Charge reversal; fiber assembly	MMP-2→GSH/GILT/lysosome	Cellular uptake, lysosomal retention, and pyroptosis; Pyroptosis inducer ± anti-PD-L1; Efficacy n = 5; immune assays n = 3	4T1; Pan02	fibers 30 min–6 h in vitro; bio-TEM at 24 h in vivo; Evidence: Direct	[63]
DEVD-DLPA@C3 (peptide NP)	~210 nm spheres → nanofiber; Fiber assembly; polymerization	Caspase-3/mtROS/mitochondria	Mitochondrial retention and localized photodynamic activity; C3; PDT; Efficacy n NR	4T1	fiber emergence 1.5 h; predominant by 8 h in vitro; Evidence: Indirect	[60]

Note: Reported times are study-specific observations under the stated conditions and are not directly comparable kinetic constants. Direct evidence denotes structural visualization in tumor tissues, whereas fluorescence retention or functional changes alone are classified as indirect evidence. NR, not reported. Unless otherwise stated, efficacy values reproduce primary reports; absence of matched loading, charge, or ligand-density information limits attribution of the entire effect to size transformation alone. Abbreviations: ADR, adriamycin-resistant; AuNP, gold nanoparticle; BB94, batimastat; BLZ945, colony-stimulating factor 1 receptor inhibitor; bio-TEM, biological transmission electron microscopy; CBT, 2-cyanobenzothiazole; Cys, cysteine; DOX, doxorubicin; ECM, extracellular matrix; GILT, gamma-interferon-inducible lysosomal thiol reductase; GSH, glutathione; HCQ, hydroxychloroquine; MMP, matrix metalloproteinase; mtROS, mitochondrial reactive oxygen species; NLG919, indoleamine 2,3-dioxygenase pathway inhibitor; NP, nanoparticle; NR, not reported; PDA, polydiacetylene; PD-L1, programmed death-ligand 1; PDT, photodynamic therapy; RT, radiotherapy; TEM, transmission electron microscopy; TME, tumor microenvironment.

**Table 3:** Multistage assembly-disassembly and disassembly-reassembly platforms.

Platform/Type	Size/Structural Trajectory	Trigger/Site	Delivery Function/Cargo/Modality	Model	Reported Time/In Vivo Evidence	Ref.
s(DGL)n@Apt (polymer NP)	104 nm NP → ~1 μm aggregate → <10 nm DGL; Enlargement; cross-link cleavage	Hypoxia/PDAC TME	Transient retention followed by deep tumor penetration; pGem + HJC0152; Efficacy n = 8; immune assays n = 3	Pan02	Hypoxia-gradient states demonstrated; formal rate NR; Evidence: Indirect	[49]
P-CyPt (small molecule)	Small molecule → ~160 nm NP → small molecules; Assembly→ disassembly	ALP → GSH/membrane → cytosol	Tumor penetration, local retention, and intracellular release; Pt(IV) prodrug; NIR/PA; Efficacy n = 4	HeLa; HepG2	GSH-mediated conversion complete within 60 min in vitro; Evidence: Indirect	[65]
Dual-NP (polymer NP)	169 nm dual-NP → 14.5 nm NP + nanogel; Disassembly; nanogel reassembly	MMP-9 + acidic pH/GBM TME	Deep penetration combined with local depot retention; DOX- dendrimer; Retention n = 5; survival n = 6–12	GL261	nanogel formation 21.8 ± 1.8 s after activation; upstream cleavage 8–48 h; Evidence: Indirect	[66]
PP NP (peptide NP)	170 nm NP → <30 nm reassembled NPs; Disassembly → reassembly	Acidic pH/extracellular TME	Sequential penetration, cellular uptake, and intracellular release; DOX prodrug + NIR dye; Efficacy n = 3	4T1	small structures observed at 8 h, pH 6.5 in vitro; Evidence: Indirect	[81]

Note: Reported times are study-specific observations under the stated conditions and are not directly comparable kinetic constants. Direct evidence denotes structural visualization in tumor tissues, whereas fluorescence retention or functional changes alone are classified as indirect evidence. NR, not reported. Unless otherwise stated, efficacy values reproduce primary reports; the absence of matched loading, charge, or ligand-density information limits attribution of the entire effect to size transformation alone. Abbreviations: ALP, alkaline phosphatase; Apt, aptamer; DGL, dendrigraft poly-L-lysine; DOX, doxorubicin; GBM, glioblastoma; GSH, glutathione; HJC0152, signal transducer and activator of transcription 3 inhibitor; MMP, matrix metalloproteinase; NIR, near-infrared; NP, nanoparticle; NR, not reported; PA, photoacoustic; PDAC, pancreatic ductal adenocarcinoma; pGem, gemcitabine monophosphate; Pt(IV), platinum(IV); TME, tumor microenvironment.

**Table 4:** Representative tumor-model microenvironmental features relevant to biomarker-responsive nanoplatform design.

Model	Reported Microenvironmental Features	Design Relevance	Ref.
4T1 syngeneic	Acidic tumor regions, hypoxia, and MMP-2/9-associated invasive behavior have been reported; these features are spatially heterogeneous.	May inform pH-, hypoxia-, or MMP-responsive activation, with thresholds validated in the specific implantation setting.	[57,78,82]
MDA-MB-231 xenograft	Direct tissue-slice measurements showed regional extracellular acidosis; hypoxia-associated MMP-2/9 regulation and invasion have also been reported.	May inform pH- and hypoxia/MMP-responsive designs while accounting for core-periphery heterogeneity.	[82,83]
B16F10 syngeneic	Substantial tumor hypoxia and hypoxia-associated metastatic behavior have been reported, with hypoxia differing from that observed in MC38 tumors.	May inform hypoxia- or acidity/MMP-responsive activation after model-specific confirmation.	[84]
CT26 syngeneic	Pimonidazole and [18F] FMISO studies demonstrate measurable, treatment-responsive intratumoral hypoxia.	May inform hypoxia-responsive activation and evaluation of treatment-induced changes in the trigger environment.	[85]
MC38 syngeneic	Hypoxia is detectable but can be lower than in B16F10 and varies with tumor region and treatment.	Supports model-specific calibration rather than transfer of a hypoxia threshold from a more hypoxic model.	[84,85]
Pan02/Panc02 syngeneic	Dense stromal features, hypoxia-responsive delivery conditions, and MMP-9-dependent matrix remodeling have been reported.	May inform hypoxia-responsive disassembly and MMP/stroma-responsive penetration strategies.	[49,86]
KPC genetically engineered	Progressive pimonidazole-positive hypoxia, HIF-1α signaling, desmoplasia, and an acidic glycolytic milieu have been reported.	May inform hypoxia- and acidity-responsive systems, with disease stage treated as a design variable.	[87]
GL261 orthotopic syngeneic	Invasive, acidic, and MMP-9-responsive conditions are used in this model; local retention and transformation depend strongly on intracranial location.	May inform locally administered MMP/pH-responsive depots, with brain-compartment validation.	[66]
U87/U87MG xenograft	Large hypoxic regions and MMP-2-associated invasive behavior have been reported in xenografts and hypoxic cultures.	May inform hypoxia- and MMP-2-responsive systems, while recognizing the limitations of human xenografts.	[88]
H22 syngeneic	MMP-2-responsive peptide transformation, tumor retention, and metastatic behavior were demonstrated in the reviewed H22 study.	May inform MMP-2-responsive assembly, but the trigger level should be measured in the specific tumor batch.	[79]
Lewis lung carcinoma/3LL syngeneic	Tumor hypoxia and MMP-2/9 activities increase with growth and correlate with metastatic efficiency.	May inform combined hypoxia- and MMP-responsive designs, with tumor size included in calibration.	[89]
HeLa xenograft	Hypoxia-associated extracellular acidification and ALP-dependent activation have been demonstrated in HeLa-derived systems.	May inform acidity- or ALP-responsive assembly, with separate validation of extracellular and cell-surface triggers.	[65]

Note: This table summarizes features reported in the representative tumor model literature to guide stimulus selection, not to recommend a single model or to define universal activation thresholds. Profiles vary with implantation site, tumor size, disease stage, sampling region, and analytical method; therefore, observations from different studies should not be interpreted as directly comparable quantitative values. MMP, matrix metalloproteinase.

## Data Availability

No new data were created or analyzed in this study.

## Abbreviations

The following abbreviations are used in this manuscript:

1-MT	1-Methyltryptophan
ALP	Alkaline phosphatase
AFM	Atomic force microscopy
CABT	2-Cyano-6-aminobenzothiazole
CDDP	Cisplatin
DLS	Dynamic light scattering
DOX	Doxorubicin
ECM	Extracellular matrix
FRET	Förster resonance energy transfer
GBM	Glioblastoma
GSH	Glutathione
HCQ	Hydroxychloroquine
HUNP	Hydrophobic core-tunable ultra-pH-sensitive nanoprobe
IDO	Indoleamine 2,3-dioxygenase
MMP	Matrix metalloproteinase
MRI	Magnetic resonance imaging
mtROS	Mitochondrial reactive oxygen species
NIR	Near-infrared
NP	Nanoparticle
PA	Photoacoustic
PAMAM	Polyamidoamine
PDA	Polydiacetylene
PDAC	Pancreatic ductal adenocarcinoma
PD-L1	Programmed death-ligand 1
PDT	Photodynamic therapy
PEG	Polyethylene glycol
PTT	Photothermal therapy
ROS	Reactive oxygen species
RT	Radiotherapy
TEM	Transmission electron microscopy
TK	Thioketal
TME	Tumor microenvironment
TPP	Triphenylphosphonium
TUNEL	Terminal deoxynucleotidyl transferase dUTP nick-end labeling
UCST	Upper critical solution temperature
